# The genomes of *Scedosporium* between environmental challenges and opportunism

**DOI:** 10.1186/s43008-023-00128-3

**Published:** 2023-12-04

**Authors:** Francesco Venice, Federica Spina, Domenico Davolos, Stefano Ghignone, Giovanna Cristina Varese

**Affiliations:** 1 Department of Life Sciences and System Biology, University of Turin, Viale Mattioli 25, 10125 Turin, Italy; 2 Department of Technological Innovations and Safety of Plants, Products and Anthropic Settlements (DIT), INAIL, Research Area, Via R. Ferruzzi 38/40, 00143 Rome, Italy; 3 Institute for Sustainable Plant Protection (IPSP), SS Turin-National Research Council (CNR), Viale Mattioli 25, 10125 Turin, Italy

**Keywords:** Emerging pathogens, Genome evolution, Extremophiles, RNA-seq, Antifungals

## Abstract

Emerging fungal pathogens are a global challenge for humankind. Many efforts have been made to understand the mechanisms underlying pathogenicity in bacteria, and OMICs techniques are largely responsible for those advancements. By contrast, our limited understanding of opportunism and antifungal resistance is preventing us from identifying, limiting and interpreting the emergence of fungal pathogens. The genus *Scedosporium* (Microascaceae) includes fungi with high tolerance to environmental pollution, whilst some species can be considered major human pathogens, such as *Scedosporium apiospermum* and *Scedosporium boydii*. However, unlike other fungal pathogens, little is known about the genome evolution of these organisms. We sequenced two novel genomes of *Scedosporium aurantiacum* and *Scedosporium minutisporum* isolated from extreme, strongly anthropized environments. We compared all the available *Scedosporium* and Microascaceae genomes, that we systematically annotated and characterized *ex novo* in most cases. The genomes in this family were integrated in a Phylum-level comparison to infer the presence of putative, shared genomic traits in filamentous ascomycetes with pathogenic potential. The analysis included the genomes of 100 environmental and clinical fungi, revealing poor evolutionary convergence of putative pathogenicity traits. By contrast, several features in Microascaceae and *Scedosporium* were detected that might have a dual role in responding to environmental challenges and allowing colonization of the human body, including chitin, melanin and other cell wall related genes, proteases, glutaredoxins and magnesium transporters. We found these gene families to be impacted by expansions, orthologous transposon insertions, and point mutations. With RNA-seq, we demonstrated that most of these anciently impacted genomic features responded to the stress imposed by an antifungal compound (voriconazole) in the two environmental strains *S. aurantiacum* MUT6114 and *S. minutisporum* MUT6113. Therefore, the present genomics and transcriptomics investigation stands on the edge between stress resistance and pathogenic potential, to elucidate whether fungi were pre-adapted to infect humans. We highlight the strengths and limitations of genomics applied to opportunistic human pathogens, the multifactoriality of pathogenicity and resistance to drugs, and suggest a scenario where pressures other than anthropic contributed to forge filamentous human pathogens.

**Supplementary Information:**

The online version contains supplementary material available at 10.1186/s43008-023-00128-3.

## INTRODUCTION

Recent estimates suggest that fungal diseases may kill more than 1.6 million people each year (Rokas 2022). This requires urgent attention, as evidence is suggesting us in different settings that climate change (Nnadi and Carter 2021) and environmental pollution (Gkoutselis et al. 2021) might increase the emergence of resistant fungal pathogens. Yet, fungi are mostly investigated for their impact on agriculture, food (Doehlemann et al. 2017; Zhang et al. 2021) and technological processes, and even in these cases they are neglected, compared to bacteria, viruses and other eukaryotes (Stop neglecting fungi, 2017). Clinical and environmental mycology do not communicate often, even if pathogenic abilities are likely trained in the environment. Indeed, most fungal human pathogens are opportunistic: their ecological success is not dependent on host infection, their growth in the human body is occasional, and often a consequence of immunodeficiency. It is therefore probable that pathogenicity traits were shaped pressures other than those present in the human body.

The spectrum of fungal species capable of colonizing the human body covers nearly all ecological guilds (Rokas 2022), including soil saprotrophs (Steffan et al. 2020; Wang et al. 2020; Mouhajir et al. 2020), plant symbionts (Mukherjee et al. 2013; Louis et al. 2014), air and sea-borne fungi (Sandoval-Denis et al. 2016; Hattab et al. 2019), and even lignocellulose-degrading macromycetes (Correa-Martinez et al. 2018; Cavanna et al. 2019; Giancola et al. 2021). The association between extremophilic lifestyle and opportunism towards humans is frequent (Gostinčar et al. 2018), suggesting that the study of evolutionary scenarios would help to better understand fungal human pathogens. Firstly, the hypothesis that highly anthropized and polluted ecosystems- where the selective pressure is strong, and the contact with humans is frequent- are driving the selection of opportunists. Secondly, the existence of a gene set challenged by both environmental stress and the human body is suggested (Gostinčar et al. 2018), culminating in the pre-adaptation of environmental fungi to colonize the human body (Rokas 2022). In this context, genomics has already proven to be an excellent tool to understand opportunism in well-known fungal genera, such as *Aspergillus* (Barber et al. 2021), *Fusarium* (Zhang et al. 2020) and *Candida* (Welsh et al. 2021).

We focused on the genus *Scedosporium* (Microascaceae)*,* increasingly recognized as a life-threatening group of fungi worldwide (Liu et al. 2019), for which genomic data is currently incomplete and fragmentary. Indeed, before the present study, genomic data for this family lacked gene models and functional characterization. The experimental design included systematical annotation and characterization of the publicly available genomic sequences, and was complemented by the addition of two novel genomes of *S. aurantiacum* MUT6114 and *S. minutisporum* MUT6113, isolated from tannery wastewaters and a Polycyclic Aromatic Hydrocarbon (PAH)-contaminated soil, respectively. The newly sequenced strains have unknown pathogenic potential, but were isolated from extremely polluted environments: following the current knowledge that extremophily and pathogenicity traits belong to a common core (Gostinčar et al. 2018), we compared their genomes with those of *Scedosporium* major pathogens, and Microascaceae in general. The study aimed at providing the first comparative framework for this family. Even though the present paper was not only focused on clinically-relevant strains, the analysis included a wide taxonomic diversity (both pathogens and non-pathogens) in order to better frame the ecophysiological traits of this understudied Family. By scaling the comparison up to 100 pathogenic or non-pathogenic ascomycetes, we addressed two main biological questions: (a) can a mere quantification of commonly described genomic traits (such as effectors and degrading enzymes) differentiate between pathogenic and non-pathogenic strains? and (b) can recently or anciently acquired genomic traits explain the adaptation to the anthroposphere? Pollution with azoles and other xenobiotics is thought to promote the emergence of pathogenic and multidrug-resistant fungi (van Rhijn and Bromley 2021; Stevenson et al. 2022). Therefore, we also used RNA-seq to evaluate whether the highlighted features can support a transcriptional response of the strains when they are challenged by a stress such as an azole-based antifungal compound.

## MATERIALS AND METHODS

### Isolation of strains used in this study

*Scedosporium minutisporum* MUT6113 was isolated from a PAH-contaminated soil where benzene, toluene, three xylene isomers (BTEX), and alkanes were predominant (Venice et al. 2020), while *S. aurantiacum* MUT6114 was isolated from tannery wastewater. The characteristics of this environment were previously described (Spini et al. 2018; Tigini et al. 2019). Two additional strains, *Trichoderma lixii* MUT4171 and *Trichoderma harzianum* MUT5453, were used for comparisons in growth experiments. The first was isolated from the same soil as *S. minutisporum* MUT6113, and the latter from landfill leachate. Dilution plating was used to isolate the strains. *Scedosporium minutisporum* MUT6113 and *T. lixii* MUT4171 were isolated on minimal Czapek medium supplemented with phenanthrene at 200 mg/L as the sole carbon source. The isolation of *S. aurantiacum* MUT6114 and *T. harzianum* MUT5453 was performed on a wastewater agarized medium as previously described (Tigini et al. 2019). Mycelium for DNA extraction was grown on 2% malt extract agar at 25 °C for 7 days. The strain is deposited at Mycotheca Universitatis Taurinensis (MUT, https://www.tucc-database.unito.it/collection_menu/1) of the Department of Life Sciences and Systems Biology, University of Torino, Torino (Italy).

### DNA extraction, sequencing, and assembly of genomes

The QIAamp DNA Microbiome Kit (Qiagen, Germany) was used to extract total DNA from mycelial samples. DNA quality and concentration were assessed with Nanodrop 2000 (Thermo Fisher Scientific). Library preparation and sequencing was done by Eurofins Genoma Group (Rome, Italy), using paired-end Illumina genomic libraries (2 × 250 bp) prepared with MiSeq v3 reagents (600 cycles).

At first, we assessed library quality with fastQC v0.11.9 (Andrews et al. 2012). Quality trimming and removal of adapters was done with Trimmomatic v0.38 (Bolger et al. 2014) (up to 3 mismatches, 20 and 8 as palindrome and simple clip threshold for adapters, respectively). The cleaned reads (91.31% and 93.2% of the original MUT6113 and MUT6114 libraries, respectively) were assembled with MEGAHIT v1.2.9 (Li et al. 2015). Contigs were ordered with MEDUSA v1.6 (Bosi et al. 2015), using the genome of *S. aurantiacum* strain WM 09.24 (NCBI accession: JUDQ01000001.1) as a backbone, then joined and filled with GapFiller v2.1.2 (Nadalin et al. 2012). The processed genome assemblies had 2,903 scaffolds for *S. aurantiacum* MUT6114 and 2,089 scaffolds for *S. minutisporum* MUT6113, and L50 values calculated with BBTools v38.95 (https://sourceforge.net/projects/bbmap/) were approximately 83 and 97 Kbp, respectively.

### Generation of gene models and dataset construction

Along with *S. aurantiacum* MUT6114 and *S. minutisporum* MUT6113, we generated gene models for the publicly available genomes of *S. apiospermum* HDO1 (Morales et al. 2017), *S. aurantiacum* WM 09.24 (Pérez-Bercoff et al. 2015), *S. boydii* IHEM 23826 (Duvaux et al. 2017), *S. dehogii* 120,008,799–01/4 (Shiller et al. 2021), Sce*dosporium* sp. IMV 00882 (Singh et al. 2017), and *Scopulariopsis brevicaulis* LF580 (Kumar et al. 2015). Gene models for *S. apiospermum* IHEM14462 (Vandeputte et al. 2014) and *Lomentospora prolificans* JHH-5317(Luo et al. 2017) were already available*.* Novel gene models were predicted with BRAKER v2.1.6 (Brůna et al. 2021) using the whole UniProt (https://www.uniprot.org/) set of ascomycetes proteins (as of June 2021) as physical evidence to model exons. The pipeline was run in “epmode” using DIAMOND 0.9.24124 for the removal of redundant hints and ProtHint 2.6.0 (Brůna et al. 2020) for the actual hints generation. The annotations were performed in “softmasking” mode (see Transposable elements identification below). A first estimation of the accuracy and completeness of the annotations was obtained with the etraining script in AUGUSTUS 3.4.0 (Stanke et al. 2008). For each genome, the proteome was then predicted from the gene models and checked with BUSCO v5.4.3 (Manni et al. 2021) for the presence of orthologous gene sets common to all ascomycetes. The percentages of ascomycete BUSCOs for the generated proteomes ranged from − 96 to − 99% and are reported in Additional file 1.

Along with the 10 Microascaceae proteomes, we selected another 90 ascomycetes for comparative purpose. The proteomes were downloaded from the NCBI and Mycocosm platforms (https://mycocosm.jgi.doe.gov/) and basic genome parameters, such as genome sizes, number of protein-coding genes, and G+C content were calculated with bash scripts and BBTools. The selected proteomes are reported in Additional file 2. According to literature data, pathogenicity can be defined as the ability of a fungus to cause damages to host tissues, while virulence refers to the amount of damage a pathogenic fungus can cause (Siscar-Lewin et al. 2019). Since information about pathogenic potential and ability to colonize human tissues is largely lacking at the strain level, we based our classification at the species level, defining different categories based on how frequently a species is found growing in the human body, and/or as a cause of damages towards the host. The definitions “potentially pathogenic”, “potentially capable of growing in the human body” and “found in clinical samples” are used throughout the text to remark this lack of information. For example, even if the pathogenicity of *S. aurantiacum* MUT6114 and *S. minutisporum* MUT6113 is unknown, these two species are frequently isolated from lung tissues of patients with cystic fibrosis (Shiller et al. 2021), and we therefore defined them as “frequently associated with clinical samples”. The definition of “rarely found in clinical samples” was reserved to fungi for which a single case or a few cases of infections in humans have been reported: for example, *Trichoderma* spp. can sporadically infect patients with strong immunodeficiency (Guarro et al. 1999), but these reports are outnumbered by those regarding other species, such as *Aspergillus*, *Fusarium* or *Scedosporium*, which can also cause nosocomial outbreaks (Vonberg and Gastmeier 2006; Cortez et al. 2008; Arnoni et al. 2018). Where possible, we also relied on current legislation about the risks for human health attributed to fungal species (https://www.who.int/publications/i/item/9789240060241). Model ascomycete human pathogens are in the genus *Candida* (Kim and Sudbery 2011), but we decided to exclude yeasts to focus mainly on fungi with predominant filamentous growth, limiting the impact of dimorphism-related genes in comparative analyses, and prioritizing understudied taxa.

### Functional annotations and quantification of specific features

Some of the 100 proteomes already had functional annotations, but we decided to re-annotate all of the dataset also to increase consistency. At first, the 100 proteomes were subjected to functional characterization using InterProScan v5.38.76 with all applications enabled. We then used Predector v1.2.6 (Jones et al. 2021) to annotate secreted proteins, effectors and Carbohydrate Active Enzymes (CAZymes). Predector is a fully automated pipeline that gathers the results of tools as TargetP, SignalP, TMHMM, EffectorP and HMM searches in the dbCAN database (Yin et al. 2012; Sperschneider et al. 2016; Almagro Armenteros et al. 2019; Hallgren et al. 2022; Teufel et al. 2022) and ranks them using a “learning-to-rank” machine learning approach. The annotation results were loaded in R v4.1.0 for summarization and subsequent analyses. For each fungus, we counted the number of proteins in specific functional categories (Additional file 2). Features such as secreted proteins, effectors and CAZymes were parsed directly from the Predector output. For other functional categories, we relied on Pfam annotations obtained with InterProScan. The full list of Pfam identifiers that were searched to quantify each functional category are reported in Additional file 3. To normalize for the difference in size between the investigated proteomes, we then transformed the counts to proportional, indicating the percentages of genes in a functional category over the size of the whole proteome. We then used a machine learning approach to find whether any of the quantified features could be deemed as a marker feature to discriminate potentially pathogenic fungi. We applied Boruta v7 (Kursa and Rudnicki 2010) with default parameters on the transformed count table for this purpose, setting a *p-*value threshold of 0.01, “6113” as seed number, and using the “TentativeRoughFix” function after the iterative discarding of unimportant features. In this context, we found that the tripartite classification of “rarely”, “frequently” and “never” associated with clinical samples added too much complexity and statistical noise to the analysis. We therefore aggregated the “rarely” and “never” categories since reasonably, a potentially pathogenic fungus is more likely to carry determinants of adaptation to the human body, rather than the two other fungal groups. The histogram plot and the dot plot were generated with ggplot2 (Wickham 2009) in the R environment.

### Phylogenomic and genome-wide analysis of variants

We built the phylogenomic reconstruction starting with the same organisms used in the previous comparison (Additional file 2). We used OrthoFinder v2.5.4 to infer orthology between the proteomes, applying DIAMOND as the homology search engine. Gene trees were produced through multiple sequence alignments using MAFFT v7 (Katoh and Standley 2013) and FastTree v2.1 (Price et al. 2010). The reconciled species tree was generated from single-copy orthologs and accounted for 64,810 individual sites. The tree was then made ultrametric with r8s (Sanderson 2003), constraining the Microascaceae node between 171 and 241 MYA, the Onygenales node between 83 and 193 MYA, the Eurotiales node between 73 and 169 MYA, and the Dothideomycetes crown between 93 and 252 MYA (Prieto and Wedin 2013; Hyde et al. 2020). Rates and times were estimated with the penalized likelihood and truncated Newton methods. Expansions and contractions in gene families were detected using CAFE 5 (Mendes et al. 2020), after the selection of an error model to account for errors in the assemblies. For each group of orthologous genes, i.e. each family, CAFE 5 reported how many members were detected at each node and tip of the tree. This way, the speed at which a family increases or decreases its members was estimated, and fast-evolving gene families were identified by assignment of a *p*-value (a 0.05 threshold was applied). For the marker features selection analysis, the output table with tips-specific increases or decreases was filtered to only keep fast-evolving gene families. Then, machine learning with Boruta was applied to select marker features to differentiate tips (species) that are frequently associated with clinical samples, from those that are not. The main phylogenetic tree and the heat trees were drawn in R using ggtree v3.5.0.901 (Yu 2020). The data required for heat trees was parsed in R by using the CAFE output, merged with the InterPro annotations of each strain.

A genome-wide variant screening analysis was carried out to closely compare *S. aurantiacum* WM 09.24 and *S. aurantiacum* MUT6114. Quality and adapter-trimmed genomic reads of *S. aurantiacum* MUT6114 were mapped against the reference *S. aurantiacum* WM 09.24 genome with bwa-mem 2.2.1 (Vasimuddin et al. 2019). The resulting BAM file was filtered to retain only mapped reads, sorted and indexed with SAMtools 1.16.1 (Li et al. 2009), and then processed with the GATK v4.3.0.0 (Van der Auwera and O’Connor 2020) pipeline to detect duplicates and correct per-base quality scores (MarkDuplicatesSpark and BaseRecalibrator, respectively). Variants were called with HaplotypeCaller and filtered with VariantFiltration, using the formula "DP < 10.0 || DP > 150.0 || GQ < 40.0". The coordinates of filtered variants were matched with those of *S. aurantiacum* WM 09.24 predicted genes with BEDTools 2.28.0 (Quinlan and Hall 2010), and InterProScan annotations were used to describe intersecting genes.

### Transposon analyses

Transposons in Microascaceae were identified starting from primary, unmasked genome sequences. The genomes were first scanned with RepeatModeler v2.0.1 (Flynn et al. 2020) (LTRharvest pipeline enabled) and TransposonPSI_08222010 (https://transposonpsi.sourceforge.net/), following a de-novo and reference-based method, respectively. After discarding sequences shorter than 50 bp, the two newly generated libraries were characterized using RepeatClassifier, and merged using vsearch v2.15.1 (Rognes et al. 2016). The non-redundant libraries of each genome were used to identify and mask insertion sites with the -s (sensitive) mode in RepeatMasker v4.1.2-p1 (Tarailo-Graovac and Chen 2009). Transposon landscapes were generated using the Kimura’s two-parameters distance as calculated by RepeatMasker and parsing them with the Parsing-RepeatMasker-Outputs collection of tools (https://github.com/4ureliek/Parsing-RepeatMasker-Outputs). Bar plots shown were drawn in the R environment using ggplot2, and further processed manually. For the TE orthology analysis, we used SAMtools to extract, from each genome, the insertions detected by RepeatMasker and their 1000 bp upstream and 1000 bp downstream flanking regions, defined in the discussion as the genomic context of the insertions. A filter was applied a priori, such that only insertions larger than 100 bp were considered. Orthology was then calculated with OrthoFinder in nucleotide mode. Orthologous insertions common to all Microascaceae or all *Scedosporium* spp. were checked with BEDTools to see whether they intersected annotated genes in the respective genomes. The 1000 bp upstream of predicted genes were also considered as putative promoter regions, since TE insertions in promoter regions can alter the expression of the affected gene (Castanera et al. 2016). The same was done with recent, strain-specific insertions: to avoid the bias of nested insertions, recent insertions which coordinates matched those of ancient ones were discarded. The Pfam and InterPro annotations of the affected genes were converted into Gene Ontology (GO) using the tables found at http://current.geneontology.org/ontology/external2go/pfam2go and https://www.ebi.ac.uk/GOA/InterPro2GO, respectively. The set of GO terms obtained this way was summarized using REVIGO (Supek et al. 2011) with the whole UniProt database as a background, and SimRel as a measure of semantic similarity. The summarizations were drawn in R with treemap (https://CRAN.R-project.org/package=treemap). Briefly, the summarizations consist of representative, non-redundant sets of Biological Process (BP) terms, based on the semantic relationships derived from the whole GO database (http://geneontology.org/). Genome architectures were generated in R with a data binning procedure described for plant pathogens (Saunders et al. 2014).

### Growth experiments

*S. aurantiacum* MUT6114, *S. minutisporum* MUT6113, *T. lixii* MUT3171 and *T. harzianum* MUT5453 were grown on agarized media. Growth was measured at room temperature (24 ºC) and at 37 ºC as representative of the human body temperature. The capability to grow in this condition is a requirement for fungal opportunistic pathogens (Gostinčar et al. 2018). Since the human body imposes mineral-limiting conditions (especially concerning iron), we tested five media with or without the addition of microelements and glucose as sole carbon source. This helped to evaluate whether the presence of an easily accessible carbon source would strengthen the primary fungal metabolism, affecting the capability to tolerate high temperature. Medium M1 had glucose (20 g/l), NaNO_3_ (3 g/l), K_2_HPO_4_ (1 g/l) and NH_4_Cl (1 g/l) as nitrogen and potassium sources, Trace Metal Solution (TMS; ZnSO_4_ 1 g, CuSO4 0.5 g in 100 ml) and a Mineral Solution (MS: KCl 5 g, MgSO_4_ 5 g, FeSO_4_ 0.1 g in 100 ml). Medium M2 had the same composition, besides the fact that iron was removed from the MS. Medium M3 was the same as M1 but without glucose, and M4 was an M3 deprived of MS and iron. The strains were grown on these media for 27 days, and colony diameters were measured at three, six, 10, 17, 22 and 27 DPI. The related plot has been generated with ggplot2.

To stimulate a transcriptional response of the two *Scedosporium* towards different antifungals, at first we performed a growth experiment in the presence of either micafungin sodium salt (Sigma-Aldricth, Cat. SML2268-5MG), amphotericin b (acqueous solution, HyClone™; VWR, Cat. HYCLSV30078.01), fluconazole (> 98% purity; VWR, Cat. TCIAF0677-1G), or voriconazole (> 98% purity; VWR, Cat. TCV0116-100MG). This first experimental stage was necessary to assess which antifungal compound had the strongest impact on the growth of the two strains. These tests were performed at 37 ºC in microplates with growing concentrations of each substance ranging from 0.125 to 128 mg/l. The inoculum was produced by homogenizing well-grown colonies in a semi-agarized rich medium (MEA, Malt Extract Agar with 0.1% agar), and 200 µl of inoculation fluid mixed with mycelium was added to a 96-wells plate containing the substances. Absorbance was used as a measure of mycelial growth, and assessed with Infinite M200 spectrophotometer (TECAN Trading, Austria) at one, two, three, four and seven DPI. Growth inhibition was measured as an absorbance ratio (converted to percentage) between the treated samples and control samples grown in the same condition without any antifungal. At the end of the trial, mycelium of both strains was transferred. The results were rendered using ggplot2 and Rstatix packages (https://CRAN.R-project.org/package=rstatix). Statistical differences between absorbance averages were calculated with the Wilcoxon test after assessing the non-normality of the data with the Shapiro–Wilk test.

### RNA-seq experiment

To produce sufficient amounts of mycelium for RNA extraction, we scaled up the volume of growth medium to 50 ml in 250 ml flasks. Treated and control samples were grown in the presence or absence of voriconazole (1 mg/l), respectively. Both *S. minutisporum* MUT6113 and *S. aurantiacum* MUT6114, control and treated samples, were grown at 37ºC in the dark with orbital agitation. Mycelium was collected at one and four DPI by centrifugation, and immediately frozen in liquid nitrogen. RNA was extracted with the RNeasy® Plant Mini Kit (Qiagen; Cat. 74,903), and subsequently treated with Ambion™ DNase I (Cat. AM2222). To remove residual reagents and sheared DNA, RNA was further cleaned and concentrated with the RNA Clean & Concentrator-5 (Zymo Research; Cat. R1013) and brought to a final volume of 35 µl. The concentration and quality of RNA samples was checked with NanoDrop 2000, while integrity was assessed with the 2100 Bioanalyzer System (Agilent). Library preparation and sequencing was done by Eurofins Ggenoma Group (Rome, Italy). Universal Plus mRNA-Seq kit (Tecan Genomics; Cat. 0520–24) was used for library preparation (2 X 150 bp), and sequencing was performed with NovaSeq 6000. In total, we sequenced 29 RNA libraries (composed of forward and reverse reads), with a minimum of three biological replicates for control samples at each time point, and four replicates for treated samples at each time point (Additional file 4). Illumina adapters were removed with Cutadapt v3.7 (with the parameters “–anywhere”, “–overlap 5”, “–times 2”, “–minimum-length 35”, “–mask-adapter”), and quality was checked with FastQC. Since no quality issues were detected, and since quality trimming strongly impacts the accuracy of gene expression estimations (Williams et al. 2016), we didn’t filter or cut reads based on quality. Forward and reverse reads were mapped with Salmon v1.8.0 (Patro et al. 2017) on the coding sequences of *S. aurantiacum* MUT6114 and *S. minutisporum* MUT6113 in mapping-based mode. We applied the correction of G+C, positional, and compositional biases (“–seqBias", "–gcBias", "–posBias"), and used soft clipping at the beginning and end of reads when they were scored with selective-alignment. Mappings were further refined with the “–ValidateMappings” option. Raw quantifications were summarized at the gene level and imported in R with tximport v1.20.0^160^. We applied HTSFilter v1.32.0 (Rau et al. 2013) on raw quantifications to remove the noise of genes with very low expression over the samples, but the filtering threshold was as low as one count-per-gene, and we therefore applied a manual filter to genes which had fewer than 10 counts across samples. Differential expression was calculated with DESeq2 v1.32.0 (Love et al. 2014). Independent filtering and the BetaPrior DESeq2 options were used, and the *p*-value threshold was 0.05 (no filtering was applied to log2 fold changes). We performed the Principal Component Analysis (PCA) and detected top and bottom loadings with pcaExplorer v2.18.0 (https://federicomarini.github.io/pcaExplorer/). The functional enrichment analyses were done using KEGG annotations, obtained for both strains with BlastKOALA (https://www.kegg.jp/blastkoala/). Since specific pathways are present in the KEGG database for *S. apiospermum* IHEM 14462, we used them as a backbone for all the functional analyses. The differential expression trees were generated with KEGG profiles in clusterProfiler v4.3.3 (Wu et al. 2021), taking advantage of the GSEA (Subramanian et al. 2005) method for pathway enrichment. To identify differentially expressed transposons, we mapped the RNA-seq reads on the primary genomic sequences of the two strains with TopHat v2.1.1 (Kim et al. 2013) and intersected the mapping results with the insertions detected by RepeatMasker. Counts-per-insertion were obtained with HTSeq v2.0.2 (Putri et al. 2022) and processed with DESeq2 as above.

## RESULTS AND DISCUSSION

### Quantitative genomic parameters do not relate with pathogenic abilities

**Fig. 1:**
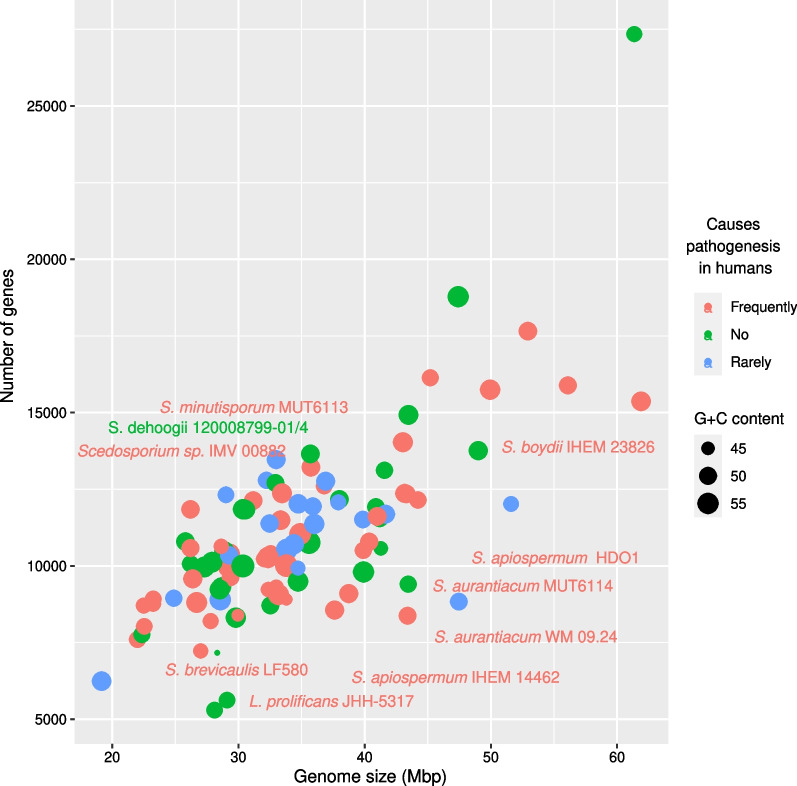
The dot plot represents the distribution of the 100 analyzed genomes in relation with number of coded genes (Y axis) and size (X axis). Each genome is represented by a dot which color is related to the frequency by which the species is found to cause pathogenesis in humans, and which size is proportional to the G+C content. No pathogen-specific distribution pattern was observed. The Microascaceae strains used in the study are highlighted

The size of the sequenced genomes fits that of other sequenced Microascaceae (including *Scedosporium*), ranging from − 33 to − 44 Mbp, and coding for − 8300 to− 12,300 genes (Additional file 2; Fig. 1). We compared these metrics with a set of another 90 Ascomycetes genomes, including putative opportunistic pathogens, occasional colonizers of human tissues, and non-pathogenic relatives. We classified each genome based on whether the species is frequently or rarely associated with damages to the human host (Siscar-Lewin et al. 2019), or whether it has never been found in clinical samples according to literature. Potentially pathogenic species didn’t show genome or gene number expansions (Fig. 1). Several bacterial human pathogens are characterized by genome size reduction and an increased G+C content which sustain DNA stability at human body temperature (Murray et al. 2021; Hu et al. 2022), but this was not true for the analyzed fungi. Potential fungal pathogens may have evolved different strategies that allow them to survive in an extreme and peculiar ecological niche, e.g. the human body. Dermatophytes, for example, rely on secreted lipases and proteases to feed on the human body (Monod et al. 2002; Park et al. 2013), while Reactive Oxygen Species (ROS) scavengers allow a broad range of systemic pathogens to cope with oxidative burst of phagocytes (Brown et al. 2009; Briones-Martin-Del-Campo 2014). Transmembrane transport and iron metabolism support fungal life at low water activity and severe iron limitation that characterize the human body (Niimi et al. 2005; Gostinčar et al. 2018). Hydrophobins are thought to be responsible for the formation of biofilm, that enhances adhesion to tissues and resistance to desiccation (Valsecchi et al. 2017), while a number of secondary metabolites have been found to be essential for virulence (Scharf et al. 2014). Since fungal genomes often undergo gene duplications to achieve a functional redundancy that supports different lifestyles, we applied machine learning to confirm whether the number of genes in these classes were selectable as markers for human pathogens, irrespective of taxonomic relationships. Glycoside hydrolases and carbohydrate esterases were deemed as marker features for pathogens, together with lipases and ROS detoxification-related genes (Additional file 5). Potential pathogens were confirmed to code for less glycoside hydrolases which is concordant with literature data (Gostinčar et al. 2018), although carbohydrate esterases followed the opposite trend. Lipases and ROS-related genes were slightly less abundant in human pathogens, which seems to contradict the evidence above.

### Large-scale phylogenomics highlights the lack of evolutionary convergence of traits that might explain colonization of human tissues

**Fig. 2:**
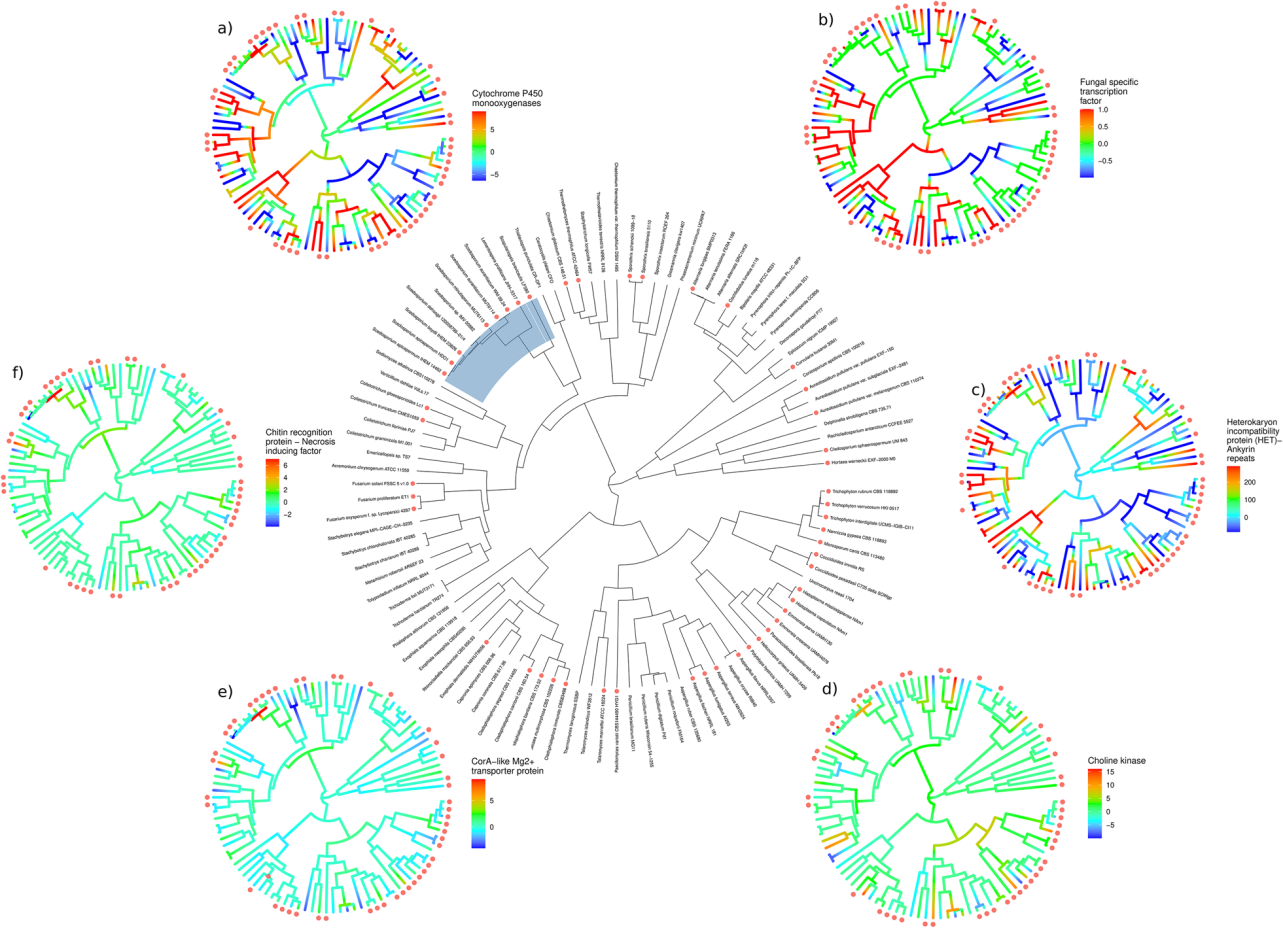
Phylogenomics reconstruction with potentially pathogenic fungi marked with a red dot in the tree (the definition of non-pathogenic includes also species that are rarely found to cause diseases in humans). The Microascaceae branch is highlighted in the main tree. The heat trees use the same phylogenomics backbone, but are color-coded based on the expansions/contractions of the specific gene families over time. The color scale gives a proportion of how many genes were lost (towards blue color) or gained (towards red color) in a gene family at each node

Since the quantification of genomic traits alone proved to be inefficient for the identification of a putative pathogenic genotype in the considered fungi, we analyzed the same data set by fitting it on a phylogenomic backbone, and thus describing evolutionary changes over time (Fig. 2). This study carefully considered the fact that even defining a human fungal pathogen is difficult: colonizing the human body is secondary for its ecological success, opportunism is homoplastic, and the spectrum of ascomycetes found in clinical samples can cover almost all families. Disseminated diseases are also different from local infections, even if both are invasive and exposed to the surveillance of the immune system (Hof 2010). The aim of the analysis was to compare a large amount of different evolutionary histories to track whether, at some point in the fungal tree of life, a selective pressure gave birth to a convergence of traits that may be linked with the ability to infect human tissues.

In general, potential opportunistic pathogens or frequent colonizers of human tissues were not characterized by faster rates of gene gains and losses when compared to environmental and plant pathogenic relatives. Since opportunists are also often able to tolerate high concentrations of pollutants (Gostinčar et al. 2018), we first focused on specific fungal gene families related with degradative processes, that might also cope with the stresses posed by growth in the human body. Among others, cytochrome P450 belong to a large class of enzymes involved in a variety of metabolic pathways: in human pathogens such as *Cladophialophora* (Herpotrichiellaceae) and *Aspergillus* (Aspergillaceae), these enzymes are particularly abundant and their role in pathogenesis and PAH degradation has been hypothesized (Kuan et al. 2016; Teixeira et al. 2017; Blasi et al. 2017). Cytochrome P450 enzymes participate in the biosynthesis of fatty acids and are the targets of azole antifungals (Warrilow et al. 2013; Yamaguchi et al. 2021). Our results indicate that P450 families evolved several times along the tree (Fig. 2a): we observed multiple and relatively ancient expansion events followed by recent contractions (and vice versa). The Herpotrichiellaceae ancestral node is characterized by a strong expansion of P450 families, and this is also true for the *Scedosporium* node. However, a contradicting scenario is observed, for example, in Arthrodermataceae, Ajellomycetaceae and *Fusarium* (Nectriaceae) opportunistic pathogens.

We used an unsupervised machine learning approach to find gene families whose expansions or contractions could characterize potentially pathogenic and non-pathogenic nodes and tips. A class of transcription factors (Fig. 2b) and others including ankyrin repeat proteins (Fig. 2c) were found as candidates. The latter has been associated with non-self recognition in fungi and might therefore be involved in the interaction with the human host (Uehling et al. 2017). However, for both families, the differences were driven by isolated cases: *Alternaria longipes*, frequently found as the agent of lung infections (Pastor and Guarro 2008), shows different patterns of evolution for the two families when compared to its closest relative *A. tenuissima*, which is very rarely found in clinical samples. Differences were also observed for clinical *vs* non-clinical (Shang et al. 2016) *Sporothrix* species and, less consistently, in the Herpotrichiellaceae clade. By analyzing a highly heterogeneous set of fungal genomes diversity can be taken into account, increasing the accuracy of phylogenomics reconstructions and allowing to precisely track the evolution of gene families (Bravo et al. 2019; Young and Gillung 2020). At the same time, increasing heterogeneity also implies decreasing the power of the analysis to connect genes and traits, as pathogenicity. A limitation of such large-scale phylogenomic analyses might be the extreme genotypic variability in fungi: indeed, a species with pathogenic potential and a non-pathogenic relative will differ in many traits other than pathogenesis.

Therefore, we restricted the focus on the *Scedosporium* clade, where *S. apiospermum* and *S. boydii* are defined as major human pathogens, *S. minutisporum* and *S. aurantiacum* are frequently isolated from the respiratory trait of patients with cystic fibrosis, while *S. dehogii* has never been found associated with human tissues (Shiller et al. 2021). The ancestral node for the genus showed an expansion in choline kinases (Fig. 2d), that are crucial determinants of pathogenicity in other microorganisms (Serrán-Aguilera 2016; Khalifa et al. 2020). As with all other clades, *Scedosporium* underwent expansion of CorA magnesium transporters (Fig. 2e) and chitin recognition genes (Fig. 2f), but in a markedly stronger way. Decaying plant matter, among the primary habitats for *Scedosporium* spp., is rich in tannins that create a magnesium-deprived environment through chelation (Klompmaker et al. 2017). *Scedosporium aurantiacum* MUT6114 was isolated from tannery wastewater, testifying to the extreme tolerance of these fungi towards tannins. Moreover, phagolysosomes of inflammatory macrophages also represent magnesium-deprived environments, and a high genomic redundancy of magnesium transporters might therefore support the growth in both plant and human tissues. CorA transporters in filamentous fungi might also support iron homeostasis (Reza et al. 2016). In pathogenic bacteria, CorA transporters have no affinity for iron (Papp and Maguire 2004). In *Magnaporthe oryzae,* they also coordinate iron homeostasis (Reza et al. 2016), and *Aspergillus fumigatus* overexpresses them in blood (Irmer et al. 2015). Secreted, chitin-binding proteins with homology to human tumor necrosis factors were well represented in *Scedosporium* (Additional file 6) and may be linked to sequestration and suppression of chitin-induced immune response (Lee et al. 2008; Kombrink and Thomma 2013; Muraosa et al. 2019), therefore representing antivirulence genes (Siscar-Lewin et al. 2019).

Since we also annotated the publicly available genome of *S. aurantiacum* WM 09.24, an environmental but highly virulent Australian strain (Pérez-Bercoff et al. 2015; Kaur et al. 2015), we wanted to compare it with *S. aurantiacum* MUT6114 at a closer level. A genome-wide genetic variant analysis (Additional file 7; Additional file 8) revealed the accumulation of variants in fungal-specific transcription factors, ankyrin repeat-containing proteins, a CorA magnesium transporter and chitin-binding genes. In addition, variants were found in genes involved in ROS-scavenging (thioredoxin, glutathione-S transferase (Staerck et al. 2019)), H_2_O_2_-mediated breakdown of complex molecules, including PAHs (GMC oxidoreductase (Harms et al. 2011)) and proteases, in particular serine proteases. It is worth noting that serine proteases are considered virulence genes in *Scedosporium* allowing for invasion of host tissues, elimination of host immunity and nutrient acquisition (Ramirez-Garcia et al., 2018). An Aur1/Ipt1-like inositol-phosphotransferase and malic enzyme carried variants between the strains. Both genes participate in lipid biosynthesis, with the former governing antifungal compounds’ susceptibility (Prasad et al. 2005; Massengo-Tiassé and Cronan 2009; Shahi et al. 2022). The presence of these variants suggests that an evolutionary pressure is exerted on these genes, and that changes in their functionality may occur in an intra-species context.

### Transposon insertions contribute to shape the Microascaceae gene space, and might be selected by evolutionary pressure

Transposable elements (TEs) contribute to rapid evolution and adaptation of fungal genomes, impacting lifestyle and promoting functional diversification (Muszewska et al. 2017, 2019). The content and importance of TE insertions in fungal genomes are often evaluated quantitatively, but their location in specific DNA regions is also relevant (Castanera et al. 2016). The genomes of plant pathogens are often invaded by TEs (Nottensteiner et al. 2018), which may co-localize with effectors in gene-sparse and fast-evolving regions to speed up the evolutionary race with plants (Dong et al. 2015). The “two-speed genome” theory has been recently revised, and this evolutionary dynamic might be now extended to non-strictly pathogenic fungi (Torres et al. 2020) with lower TE percentages, but higher number of genes affected by insertions, such as animal pathogens (Muszewska et al. 2019).

**Fig. 3:**
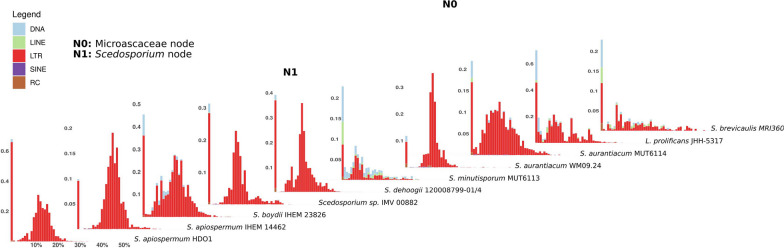
Transposons landscapes in Microascaceae genomes. The ancestral nodes for Microascaceae and *Scedosporium* have been named N0 and N1, respectively. For each strain, a histogram shows the set of insertions with their classification (color), divergence from the ancestral consensus, as calculated with the Kimura two-parameters distance (X axis) and number of base pairs over the whole genome sizes (Y axis). Divergence from ancestral sequences is used here as a measure of ancientness of the insertions

Transposons in the analyzed Microascaceae accounted for ~ 2 to ~ 6% of the total length of the genomes, with Long Terminal Repeats (LTR) retrotransposons being overall the most abundant class (Fig. 3; Additional file 9). We reconstructed, for each TE class, a consensus sequence that represents the ancestor of the observed insertions. Since TEs tend to accumulate mutations over time (SanMiguel et al. 1998; Horns et al. 2017), the sequence divergence between an insertion and its ancestor can be used as a metric to date the insertion. In Microascaceae, very recent transpositions outnumbered those with medium to high divergence from the ancestral sequences (Fig. 3). Several insertions were orthologous (i.e., they shared the same sequence and insertion site) in all *Scedosporium* or even all Microascaceae, and probably took place in their relative common ancestors. Regardless of the inferred age, all insertions in Microascaceae localized in gene-rich regions, and a non-compartmentalized architecture was observed (Additional file 10). The absence of a “two-speed genome” architecture that might contribute to a faster evolution of effectors to hijack the host’s defenses (Dong et al. 2015; Pusztahelyi et al. 2015; Selin et al. 2016; Wei et al. 2022) supports the concept that Microascaceae do not have an history of co-evolution with a human host.

A number of Microascaceae orthologous insertions overlapped genes involved in the catabolism of lipids and carbohydrates, as well as in transmembrane transport, proteolysis and DNA/RNA regulation (Additional file 11; Additional file 12). In general, alterations and/or suppression of lipids and carbohydrate catabolism have a great impact on virulence, since both the enzymes and the molecules involved constitute antivirulence factors (Siscar-Lewin et al. 2019), while proteolysis was already mentioned to be a virulence factor in *Scedosporium* invasive growth (Ramirez-Garcia et al., 2018). Additional transposition events probably took place in the *Scedosporium* common ancestor, and affected genes involved in the same cellular processes, plus iron-sulfur cluster assembly genes (Additional file 11; Additional file 12), glutathione metabolism and glutaredoxins. Of note, these components are actually considered fundamental for virulence in several pathogenic fungi, including *S. apiospermum*, governing iron sensing, homeostasis, and oxidative stress resistance (Talib & Outten, 2021; Gupta & Outten 2020; Attarian et al. 2018; Staerck et al. 2018).

We then focused on terminal branches of the Microascaceae tree: these more recent transpositions again affected lipid and steroid metabolism, proteolysis, ABC membrane transporters, DNA repair and RNA regulation (Additional file 13; Additional file 12). In *S. apiospermum* HDO1 and *S. brevicaulis* LF580, insertions involved phosphatidylinositol metabolism, but *S. apiospermum* HDO1 also had insertions in mycotoxin-related genes. Maintenance of redox homeostasis correlated with recent insertions in *S. apiospermum* HDO1 and *S. minutisporum* MUT6113, both able to tolerate high concentrations of PAHs (Morales et al. 2017). Non-orthologous transpositions in carbohydrate catabolism genes were found in all Microascaceae, with the exception of *S. dehogii* 120,008,799–01/4 and its closest relative, *Scedosporium* sp. IMV 00882. This could indicate a recent, convergent selection of insertions in different genes with the same function, as it is also evident for genes involved in cellular respiration which carry recent insertions in distant relatives of the tree (Additional file 13). Another example of this phenomenon is the insertion of transposons in the proximity of rhamnosidases, which is shared among *S. aurantiacum* MUT6114, and the major pathogens *S. boydii* and *L. prolificans*. The removal or masking of rhamnose groups from cell wall components is among the key virulence factors that allow *Scedosporium* spp. to elude the immune system, as rhamnomannans are highly immunogenic (Figueiredo et al. 2010). *Lomentospora* rhamnose conjugates also mediate the adhesion between conidia and mouse peritoneal macrophages (Xisto et al., 2015, 2019). Interestingly, we observed recent transposon insertions interesting rhamnosidases in the two pathogens *S. boydii* and *L. prolificans*, but also in *S. aurantiacum* MUT6114. Of note, rhamnose is also present in lignocellulosic biomass, a very frequent isolation source of *Scedosporium* spp. (Poirier et al., 2023; Huang et al., 2021). In our study, other glycoside hydrolases required for growth on lignocellulosic biomass were found to be impacted by recent transposition events in Microascaceae, while others seem to be reduced in other Ascomycetes with pathogenic potential. These genes require further characterization to define their unique substrates and eventually determine a potential role in virulence, as already done for α-glucans-related genes in *L. prolificans* an *S. boydii* (Xisto et al. 2023; Lopes et al. 2011).

For both phylogenomics and transposons analyses, literature gave evidence of the double role of the highlighted features, being associated with environmental stressors but also contributing to interaction and infection in the human host. This agrees with the common understanding that an “environmental school” exists, fueled by competition and stressful conditions, that might eventually exert a pressure on genes allowing colonization of the human body (Siscar-Lewin, 2022). The hypothesis of ancestral adaptation of pathogenicity traits is also supported by the evidence that several ancient fungal lineages include animal pathogens (Naranjo-Ortiz & Gabaldon, 2019). As regards proteases and glutaredoxins, they may contribute to fungal virulence (Talib & Outten, 2021; Gupta & Outten 2020; Attarian et al. 2018; Staerck et al. 2018; Ramirez-Garcia et al., 2018), but also sum up to the extraordinary degradative ability and adaptability of *Scedosporium* spp., which can thrive in very recalcitrant substrates and/or extreme environments. Of note, different (and likely recent) insertional patterns were detected in *S. aurantiacum* WM 09.24 *vs S. aurantiacum* MUT6114, involving chitin-related genes and proteases as well, probably indicating that a certain evolutionary pressure is still exerted on those features. The collected evidences should stimulate further attention on rhamnose-related genes and glycoside hydrolases, as another example of features with a dual role in environmental adaptation and virulence.

### Impact of temperature and treatment with antifungals on growth of *S. aurantiacum* MUT6114 and *S. minutisporum* MUT6113

**Fig. 4:**
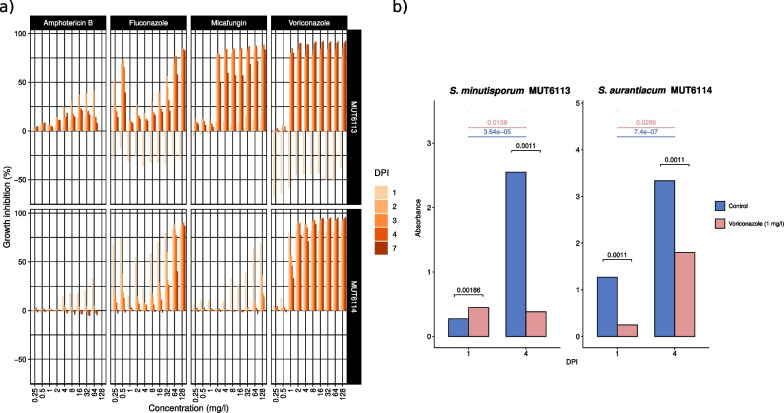
**a** Percentages of growth inhibition observed for *S. aurantiacum* MUT6114 and *S. minutisporum* MUT6113 challenged by different antifungals. Boxes are organized based on the molecules and strains. Each box shows the inhibition compared to control (Y axis) and depending on the concentration of the molecule (X axis). The color of the bars is related to the different time points of the trial. **b** Absorbance values for control samples (blue) and voriconazole-treated samples (red) in the conditions used for the RNA-seq experiment. The growth experiments were performed on microplates to monitor fungal growth spectro-photometry

We performed a transcriptomic analysis on *S. minutisporum* MUT6113 and *S. aurantiacum* MUT6114 exposed to an antifungal drug, to understand which pathways are affected by such stress, and whether these included the recently or anciently shaped features described before. We first determined experimentally the optimal growth conditions for both strains, when compared to two other non-pathogenic ascomycetes, *T. lixii* MUT3171 (Venice et al. 2020), isolated from the same PAH-contaminated soil as *S. minutisporum* MUT6113, and *T. harzianum* MUT5453 (this study), isolated from landfill leachate where temperature can typically exceed 50 ºC (Nanda and Berruti 2021). The results indicate that *S. aurantiacum* MUT6114 growth at 37 ºC is equal, if not faster, than its growth at 24 ºC (Additional file 14). *Scedosporium minutisporum* MUT6113 growth was only slightly inhibited by the human body temperature, while *T. lixii* MUT3171 and *T. harzianum* MUT5453 grew significantly slower at 37 ºC. We tested different growth media with or without the addition of minerals, including iron, but the driving factor proved to be the temperature. Literature data indicates the optimal growth temperature of *Scedosporium* spp. between 30 and 40 ºC (Guarro et al. 2006; Hoog et al., 1995), while not every strain is capable of growing at 37 ºC. Other studies found that *S. aurantiacum* and *S. minutisporum* conidia achieve 100% and 80% germination at 37 ºC, respectively (Mello et al. 2016). Highly virulent strains of *S. aurantiacum* have optimal growth temperatures between 28 and 37 ºC (Kaur et al. 2015), but *Scedosporium* spp. in general can tolerate up to 40 ºC (Chen et al. 2021), suggesting that these fungi have high plasticity in terms of growth temperature, while also pointing out that this plasticity might be strain-specific,

We treated the two *Scedosporium* strains grown in liquid malt extract over seven days with different concentrations of micafungin (echinocandin), targeting β-glucan biosynthesis, amphotericin b, fluconazole and voriconazole, targeting ergosterol biosynthesis and used against local and disseminated *Scedosporium* infections (Muñoz et al. 2000; Bosma et al. 2003; Yustes and Guarro 2005). Our aim was not to determine the degree of antifungal resistance in the two strains but to find antifungal concentrations which would produce a significant stress condition. We relied on the current knowledge about Minimum Inhibitory Concentration (MIC). According to the EUCAST (https://www.eucast.org/), the MIC of micafungin is at most 2 mg/l for pathogenic *Candida* species. The same was observed for *S. minutisporum* MUT6113, especially after three days of incubation, while *S. aurantiacum* MUT6114 could tolerate up to 128 mg/l of the substance without significant growth inhibition in the last days of the trial (Fig. 4a). Amphotericin b, whose MIC can be as high as 8 mg/l against *Aspergillus* spp., was not effective against either of the two strains, even inducing a higher late growth in *S. aurantiacum* MUT6114. The two azole drugs were the most effective against both strains, with detectable growth inhibition from 0.5 to 2 mg/l. At the end of the experiment, even after 128 mg/l voriconazole treatment, mycelium was able to reactivate its primary metabolism and grow on solid rich medium, indicating that the effect was mainly fungi-static.

### RNA-seq reveals a multifactorial response to antifungal-induced stress in *S. aurantiacum MUT6114 and S. minutisporum MUT6113*

As regards to RNA-seq trials, we chose voriconazole treatment at 1 mg/l, a concentration that would not completely inhibit the transcriptional profile of the two fungi. Notably, voriconazole is the most widely used drug in the case of disseminated *Scedosporium* infections (Morio et al. 2010; Paajanen et al. 2019; Verma et al. 2014.). We measured transcript expression at one- and four-Days Post Inoculum (DPI) since this allowed us to observe an asymmetry between the strains: *S. minutisporum* MUT6113 growth was stimulated by voriconazole treatment at one DPI, but was strongly inhibited at four DPI, while *S. aurantiacum* MUT6114 had a slighter inhibition at four DPI (Fig. 4b).

As expected, the treatment had a deep impact on the transcriptional profile of the two fungi. At all time points, steroid biosynthetic genes were up-regulated (Additional file 15), likely to counteract the inhibitory effect of voriconazole. Besides, during the experiment there was a balance between up and down-regulated genes (Additional file 15; Additional file 16), indicating no correlation between this parameter and mycelial growth. The percentage of Differentially Expressed Genes (DEGs) in response to voriconazole (over all the gene sets) was around 50%, except for *S. aurantiacum* MUT6114, with 64% DEGs at four DPI. Transmembrane RTA proteins, which are associated with resistance to toxic compounds in yeast and involved in azole resistance in *Candida (*Noble et al. 2013*)*, were among the genes that mostly contributed to differentiate control from treated samples. Likewise, several other genes associated with lipid metabolism and biofilm formation, as well as ABC transporters and antioxidant genes, had the same weight. For instance, ABC transporters have been associated with voriconazole resistance, and voriconazole itself has been shown to inhibit biofilm formation in *Candida* (Valentín et al. 2012; Gohar et al. 2017)*.* Rhamnose metabolism was also impacted by antifungal treatment in both *S. aurantiacum* MUT6114 and *S. minutisporum* MUT6113 (Additional file 15), confirming that the modulation of cell wall components is crucial in the response of these fungi to stress.

**Fig. 5:**
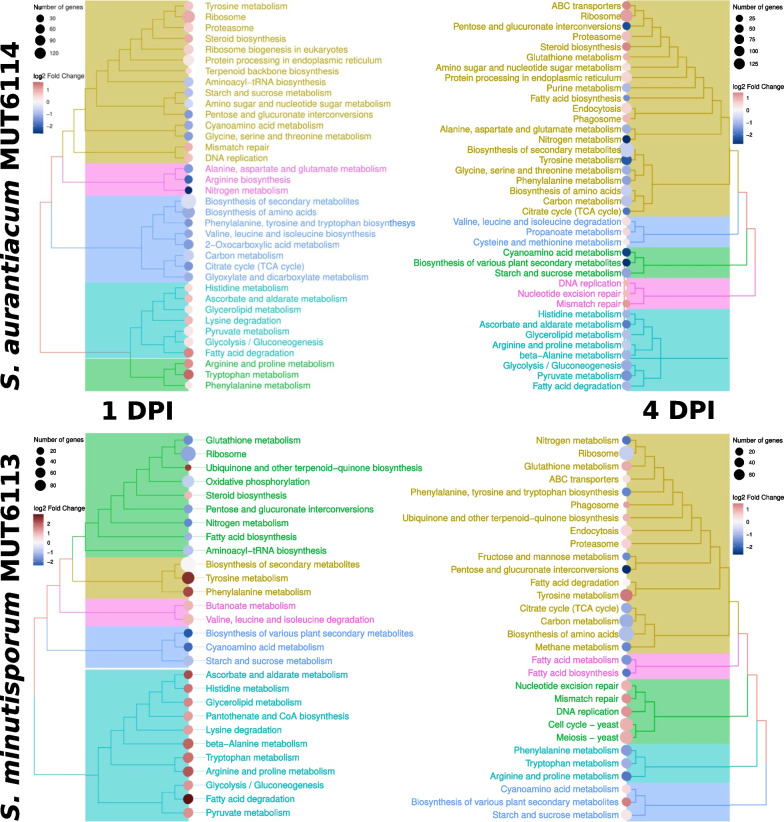
KEGG enrichments showing the main metabolic areas affected by voriconazole treatment in *S. aurantiacum* MUT6114 (top) and *S.minutisporum* MUT6113 (bottom). Each leaf (dot) of the tree represents a specific pathway, and is coloured according to the regulation of the involved genes (red for overall up-regulation and blue for overall down-regulation of the pathway). The pathways are organized in a tree structure to represent the semantic and biological overlap between them (i.e., pathways that share many genes and functions are closely related in the tree)

A broader look at the transcriptional profiles of the two fungi upon treatment (Fig. 5) revealed a partial overlap in their responses to voriconazole. Both fungi activated catabolic pathways involving amino acids, lipids and sugars, while up-regulated DNA replication was always observed. In *Candida*, the ability to catabolize lipids guarantees the pool of building blocks necessary to support membrane plasticity and resistance to azoles (Singh and Prasad 2011). The hyperactivation by both *Scedosporium* of glycolysis and glycerol metabolism at the early stages of voriconazole exposure also recalls what was observed in an azole-resistant *Candida glabrata* strain (Rogers et al. 2006). Since voriconazole has been shown to induce a moderate activation of the High Osmolarity Glycerol (HOG) pathway in *S. apiospermum* (Yaakoub et al. 2023), we also looked at the expression of the response regulator Ssk1 (g1351 and g9875 in *S. aurantiacum* MUT6114 and *S. minutisporum* MUT6113, respectively) and the key Mitogen-Activated Protein Kinase (MAPK) HOG1 (g7871 and g1735 in *S. aurantiacum* MUT6114 and *S. minutisporum* MUT6113, respectively) governing the pathway. The expression of Ssk1 did not show any significant change at any time point in both strains. By contrast, HOG1 was down-regulated in *S. aurantiacum *MUT6114 at both time points, while *S. minutisporum *MUT6113 overexpressed the gene at 4 DPI. These contrasting results might be due to the fact that the HOG response in *S. apiospermum* is activated as early as one hour after the voriconazole treatment (Yaakoub et al. 2023). In addition, the activation of HOG1 relies mainly on protein phosphorylation, rather than changes in its expression, and transcriptomics might not be the best tool to observe HOG pathway changes at these time points. The metabolism/catabolism of several amino acids might respond to nitrogen requirements imposed by the treatment: this seems to be supported by the fact that, in *S. aurantiacum* MUT6114, the proteasome pathway for protein degradation is always overexpressed, while in *S. minutisporum* MUT6113 this is true only at four DPI. Indeed, *S. minutisporum* MUT6113 grew faster upon voriconazole treatment at one DPI. Another possibility is that some amino acids act as antagonizers of azole-induced stress: for example, proline is known to mitigate oxidative stress in filamentous fungi (Li et al. 2018). In *S. aurantiacum* MUT6114, glutamate biosynthesis is down-regulated at both time points and this may lead to accumulation of its precursor amino acid, glutamine, that can fuel the steroid biosynthetic pathway upon azole treatment (Dumoulin et al. 2020). Tyrosine biosynthesis, up-regulated by both fungi at one DPI, is linked to the biosynthesis of melanins, pigments that reduce cell wall permeability and sequester antifungals in vitro, increasing antifungal resistance (Scharf et al. 2014). Glyoxalate and propanoate metabolism were differentially expressed only in *S. aurantiacum* MUT6114 and relate with peroxisomal β-oxidation of lipids upon carbon starvation and detoxification of its toxic byproducts (Sun et al. 2013; Santos et al. 2020).

The metabolic areas that are affected by genetic variants in *S. aurantiacum* MUT6114 vs *S. aurantiacum* WM 09.24, including GMC oxidoreductases, proteases and the Aur1/Ipt1-like inositol-phosphotransferase, responded to the treatment in both *S. aurantiacum* MUT6114 and *S. minutisporum* MUT6113 (Additional file 17). In *S. aurantiacum* MUT6114 and *S. minutisporum* MUT6113, 65% and 95% of genes affected by ancient transposon insertions were functional and responded to voriconazole. Concerning genes affected by recent insertions, 84% and 64% of them responded to the treatment in *S. aurantiacum* MUT6114 and *S. minutisporum* MUT6113, respectively. All of these DEGs included lipid-related, chitin-related, antioxidant and ankyrin-repeat genes, as well as CorA magnesium transporters, transcription factors and genes involved in DNA/RNA processing. Voriconazole increased the expression of 22 and 9 transposons in *S. aurantiacum* MUT6114 and *S. minutisporum* MUT6113 (Additional file 18).

The stress induced by voriconazole affected chitin and melanin-related genes. These genes were highlighted in our analyses as anciently shaped traits, and both are associated with stress resistance and adaptation (Campana et al. 2022; Dubey et al. 2020; Cordero & Casadevall 2017; Gessler et al. 2014). At the same time, chitin is highly immunogenic and chitin metabolism in *Scedosporium* and *Lomentospora* contributes to virulence and resistance to antifungals (Angiolella et al., 2022; Kitisin et al. 2021; Komi et al. 2018; Pellon et al. 2018). Melanin is also recognized as a main virulence factor in *Lomentospora* and *Scedosporium* spp. (Ramirez-Garcia et al., 2018). It could be hypothesized that a stimulus impacting the ability of a fungus to produce, bind or sequester cell wall components may also alter its pathogenic capabilities. Furthermore, these stimuli cannot be attributable solely to the interaction with the human body, as our analyses provided evidence that an ancestral selective pressure already existed and shaped *Scedosporium* and *Lomentospora* gene families.

## CONCLUSIONS

Fungal genomes hold secrets that are manifested through the extreme morphological and physiological diversification of these microbes. Our analysis on 100 fungal genomes strengthened the hypothesis that pathogenicity is characterized by a lack of evolutionary convergence at Phylum-level, and that virulence traits might have evolved multiple times along the Ascomycota tree of life. A significant convergence was instead observed in several traits at the Family and Genus level: Microascaceae and *Scedosporium* were found to share a gene pool that was both anciently and recently shaped. Indeed, the expansion of gene families, transposon insertions and point mutations date back to the respective common ancestors, and a similar path was also described for the terminal branches of the tree. What observed at the Genus level for *Scedosporium* spp. adheres to the hypothesis of an “environmental school” that might support the emergence of putative virulence and/or opportunistic traits. A major limitation of this study resides in the fact that no clinical isolates were used in the RNA-seq experiment to validate the results. Further studies will be required on a larger number of environmental and clinical isolates to validate the intertwining between environmental and virulence traits that are expressed during exposure to antifungals.

We argue that, even if some of the genetic tools for pathogenicity and antifungal resistance might have developed irrespective of anthropization, this does not limit the impact of human activities on the emergence of fungal pathogens. By decreasing biodiversity and releasing pollutants as microplastics, we boost their populations and provide vectors for their spreading, increasing exposure and infection risks. Our study will hopefully stimulate novel thoughts about fungal human pathogens and their evolution. Accumulating strain-level genomics and transcriptomics knowledge proved to be efficient to understand and identify bacterial human pathogens (Quainoo et al. 2017; Duchêne et al. 2020; Avican et al. 2021). We hope to urge novel studies on fungal human pathogens in this context, embracing the concept that a fungal pathogen is not only a harmful organism, but also a finely tuned machine whose evolutionary history is the unexplored path to its pathogenic adaptation.

### Supplementary Information

**Additional file 1.** BUSCO completeness scores for the newly-generated Microascaceae gene models.

**Additional file 2.** List of genomes selected for this study, and relative genome parameters. The table also reports whether a specific strain is frequently found to cause disease in humans, based on clinical mycology literature. The definition of “rarely found in clinical samples” was reserved to fungi for which a single case or few cases of infections in humans. The amount of proteins in specific functional areas are also reported for each strain.

**Additional file 3.** List of Pfam identifiers used to count, for the 100 analyzed genomes, the absolute number of proteins putatively involved in pathogenicity.

**Additional file 4.** Quantitative overview of the generated RNA-seq libraries, with number of total pairs (histogram plot), metadata and number of reads mapped to the respective sets of coding sequences (table).

**Additional file 5.** Histogram showing the percentage of genes in specific categories, with respect to the whole gene sets of non-pathogenic species (blue) and major human pathogens (red). The categories were selected with a feature selection algorithm.

**Additional file 6.** List of of orthologous chitin-bind gene found in an expanded family at the Scedosporium node. SignalP and TargetP scores were computed and ranked with Predector.

**Additional file 7.** GATK output including the point mutations detected between *S. aurantiacum* MUT6114 and *S. aurantiacum* WM 09.24.

**Additional file 8.** List of *Scedosporium aurantiacum* WM 09.24 genes that contain genetic variants in comparison with* S. aurantiacum* MUT6114. The annotations, as obtained with InterproScan are also shown.

**Additional file 9.** Summary of the different transposons classes predicted in the analyzed Microascaceae genome. For each class/genome, the table shows the number, total length and percentage of base pairs over the length of the entire genomes.

**Additional file 10.** Genome architecture in Microascaceagraphical representation of the growth rates of *S. aurantiacum* MUT6114, *S. minutisporum* MUT6113, *T. lixii* MUT4171 and *T. harzianum* MUT5453 on different growth media, at 24 °C and 37 °C. In each box, dots are distributed on the vertical axis depending on the diameter of the colonies at a specific time point (X axis). The lines, dots and error bars are colored based on the growth temperature. M1 to M4 indicate the different agarized media. e. The flanking distance between neighboring genes provides a measurement of local gene density and is displayed as a colour-coded heatmap, based on a whole-genome analysis. The graphics are organized in bins: each area is colored with more or less intensity based on how many genes in the genome have that 5’ (Y axis) and 3’ (X axis) distances with their neighbors. The majority of genes in Microascaceae are found in gene-dense regions (contrary to two-speed genomes). The distribution of ancestral (N0 and N1) and strain-specific insertions is also displayed with different shapes, revealing a topological overlap between TEs and gene space.

**Additional file 11.** REVIGO summary of Gene Ontology annotations for genes interested by ancestral insertions. Each rectangle is a single cluster representative. The representatives are joined into 'superclusters' of loosely related terms, visualized with different colors. Rectangles are sized relatively based on how many GO terms were found in a category.

**Additional file 12.** Genes interested by Orthologous (ancestral) and strain-specific (recent) transposon insertions. The N0 sheet contains genes with insertions that are orthologous for all Microascaceae, while N1 refers to orthologous insertions in the genus* Scedosporium*. The remainder of the sheets contains genes with strain-specific insertions. The annotations of each gene, as obtained with InterproScan, are also reported.

**Additional file 13 (a–k).** REVIGO summary of Gene Ontology annotations for genes interested by strain-specific insertions. Each rectangle is a single cluster representative. The representatives are joined into 'superclusters' of loosely related terms, visualized with different colors. Rectangles are sized relatively based on how many GO terms were found in a category.

**Additional file 14.** Graphical representation of the growth rates of *S. aurantiacum* MUT6114, *S. minutisporum* MUT6113, *T. lixii* MUT4171 and *T. harzianum* MUT5453 on different growth media, at 24 ºC and 37 ºC. In each box, dots are distributed on the vertical axis depending on the diameter of the colonies at a specific time point (X axis). The lines, dots and error bars are colored based on the growth temperature. M1 to M4 indicate the different agarized media.

**Additional file 15.** Summary of the transcriptome for *S. aurantiacum* MUT6114 and *S. minutisporum* MUT6113 in response to voriconazole. Principal Components Analyses (PCA) and a summary table are viewed at the top of the figure. The bottom area shows, for each strain and time point, the bottom and top loadings for PCA, i.e. the genes that mostly influence the distribution of dots on PC1 and PC2, and their annotations.

**Additional file 16.** Full list of Differentially Expressed Genes (DEGs) at 1 and 4 DPI for *S. aurantiacum* MUT6114 and *S. minutisporum* MUT6113. Log2 Fold Change is a logarithmic measure of the increase/decrease in gene expression. A positive log2FoldChange means that a gene is up-regulated in the treated samples, and vice-versa. Padj is an adjusted measure of the significance for an up- or down-regulation pattern.

**Additional file 17.** Starting from the previous result that specific metabolic areas are interested by genetic variations in two *S. aurantiacum* strains, the table shows whether genes in such metabolic areas are up- or down-regulated by treatment with voriconazole at 1 or 4 DPI.

**Additional file 18.** Starting from the previous result that genes in Microascaceae and *Scedosporium* are interested by ancestral (N0 and N1) and strain-specific insertions, the table shows wether these genes are among the DEGs of both strains in response to voriconazole. The up-regulation of a limited number of transposons in response to voriconazole is also reported.

## Data Availability

The genome sequences, gene models and insertions coordinates generated in this study are available at Zenodo with https://doi.org/10.5281/zenodo.7442039. The genomics and transcriptomics raw reads will be made available at NCBI upon publication, under the Bioproject accessions PRJNA514915 and PRJNA514913.

## Abbreviations

PAHs: Polycyclic Aromatic Hydrocarbons
BTEX: Three xylene isomers
CAZymes: Carbohydrate-active enzymes
MYA: Million years ago
TEs: Transposable elements
GO: Gene ontology
MS: Mineral solution
DPI: Days post inoculation
ROS: Reactive oxygen species
GMC: Glucose-methanol-choline
LTR: Long terminal repeats
MIC: Minimal inhibitory concentration
DEGs: Differentially expressed genes
