# Supplementary figures and images for: The genomes of Scedosporium between environmental challenges and opportunism
Source: IMA Fungus. 2023 Dec 4;14:25. doi: 10.1186/s43008-023-00128-3 (PMC10694956; doi:10.1186/s43008-023-00128-3)

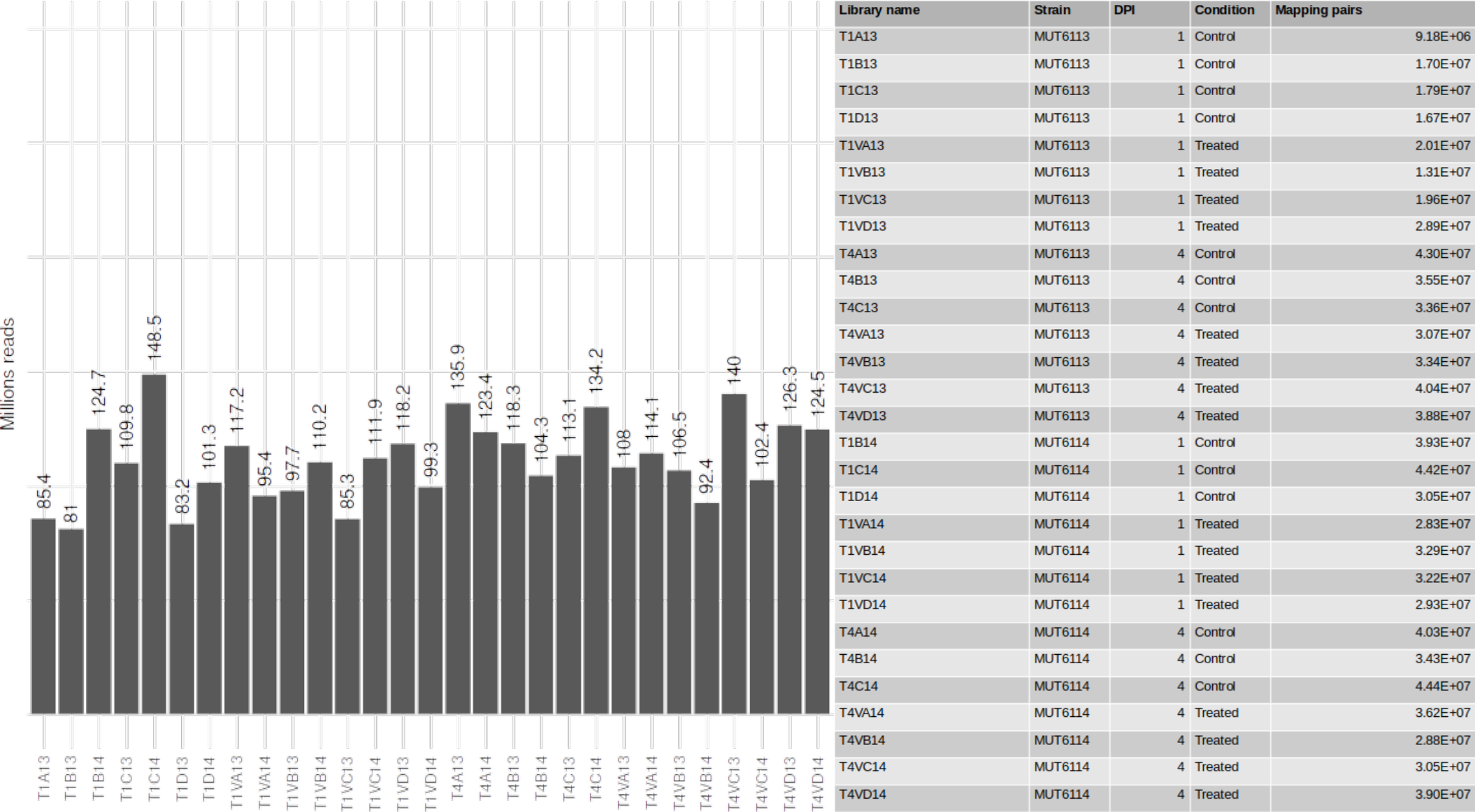

Supplement: Supplementary file 4 — Additional file 4. Quantitative overview of the generated RNA-seq libraries, with number of total pairs (histogram plot), metadata and number of reads mapped to the respective sets of coding sequences (table). [file 43008_2023_128_MOESM4_ESM.pdf]

Percentage over the whole proteomes

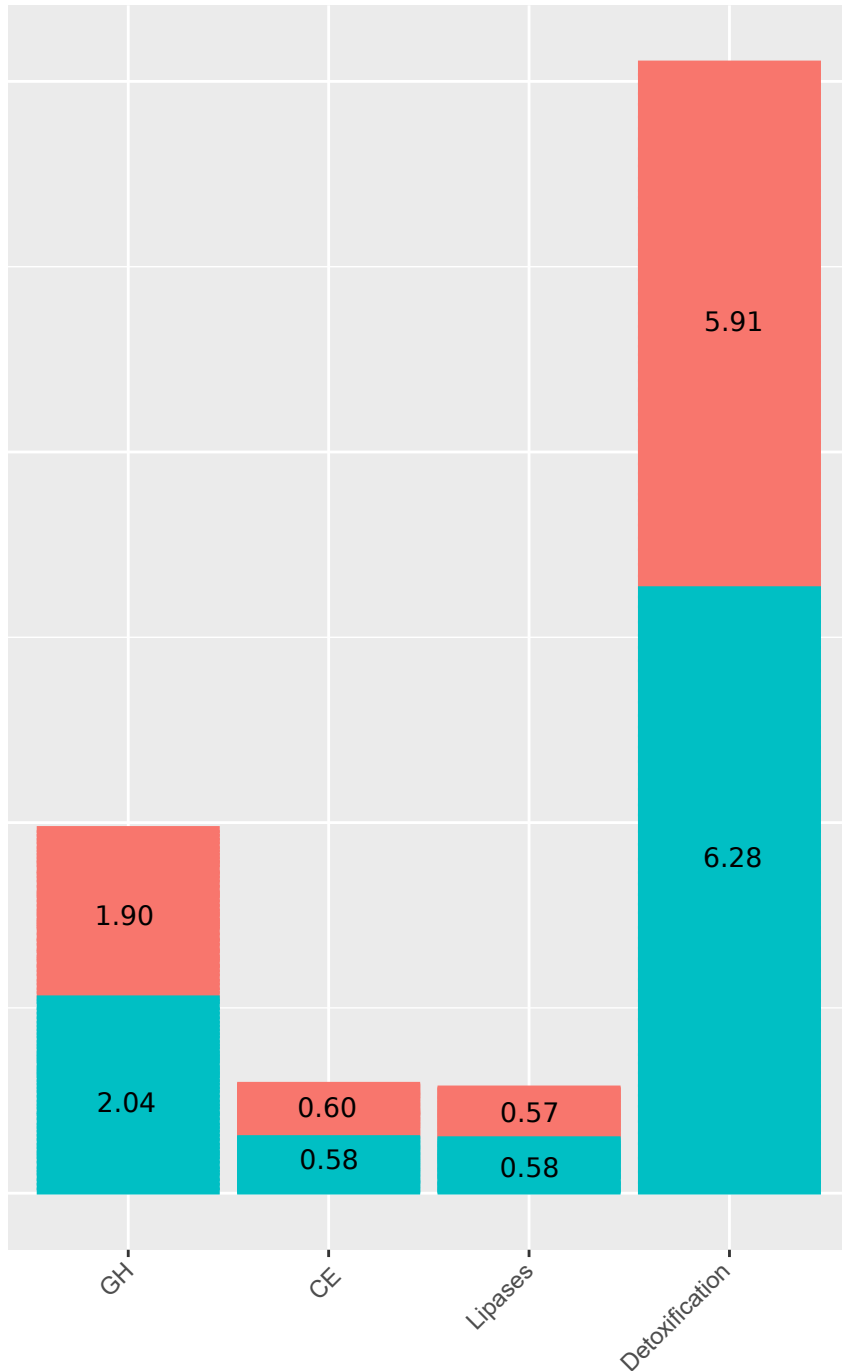

Potential human pathogen

yes

no

Supplement: Supplementary file 5 — Additional file 5. Histogram showing the percentage of genes in specific categories, with respect to the whole gene sets of non-pathogenic species (blue) and major human pathogens (red). The categories were selected with a feature selection algorithm. [file 43008_2023_128_MOESM5_ESM.pdf]

a)

N0

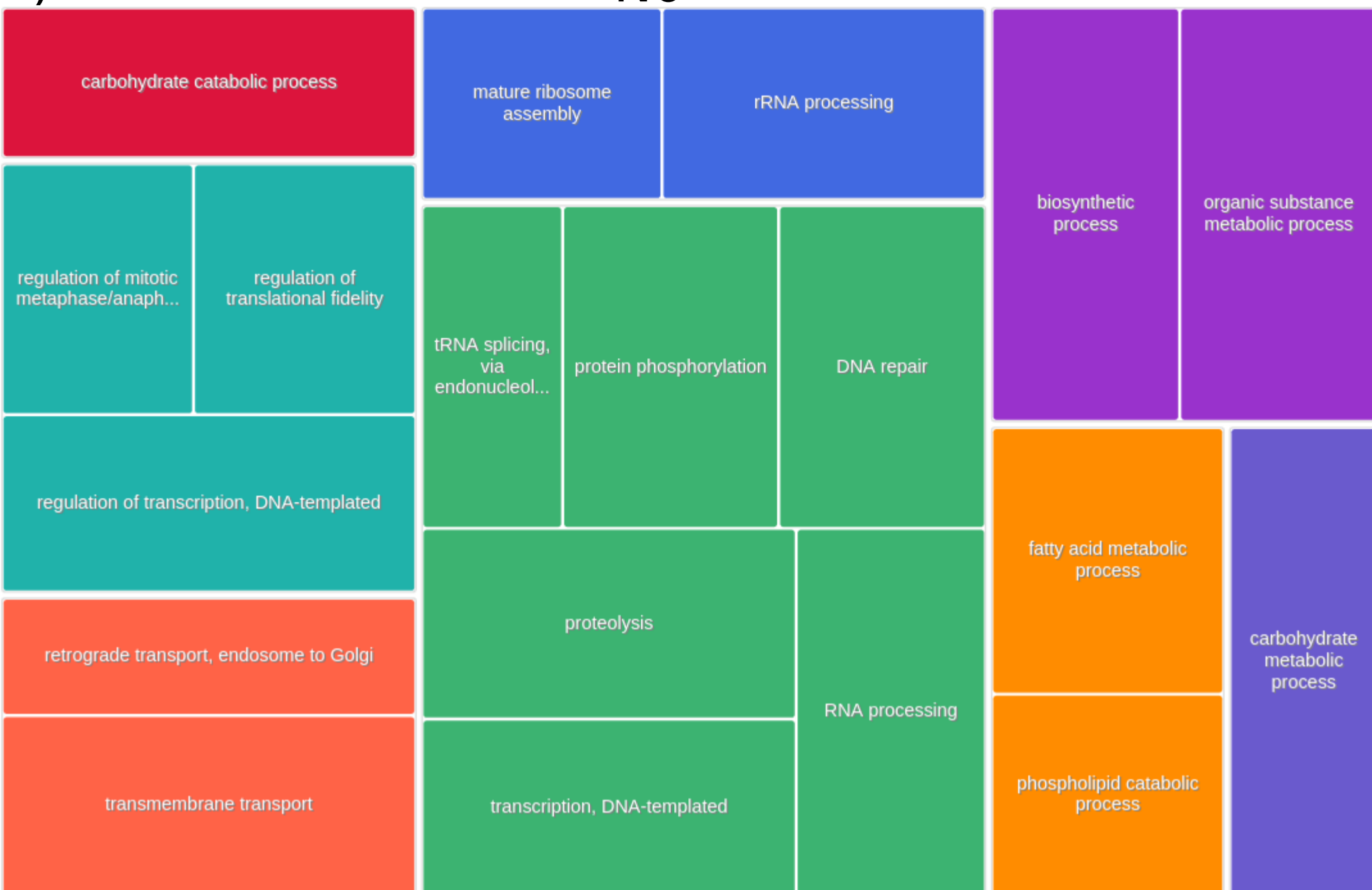

b)

N1

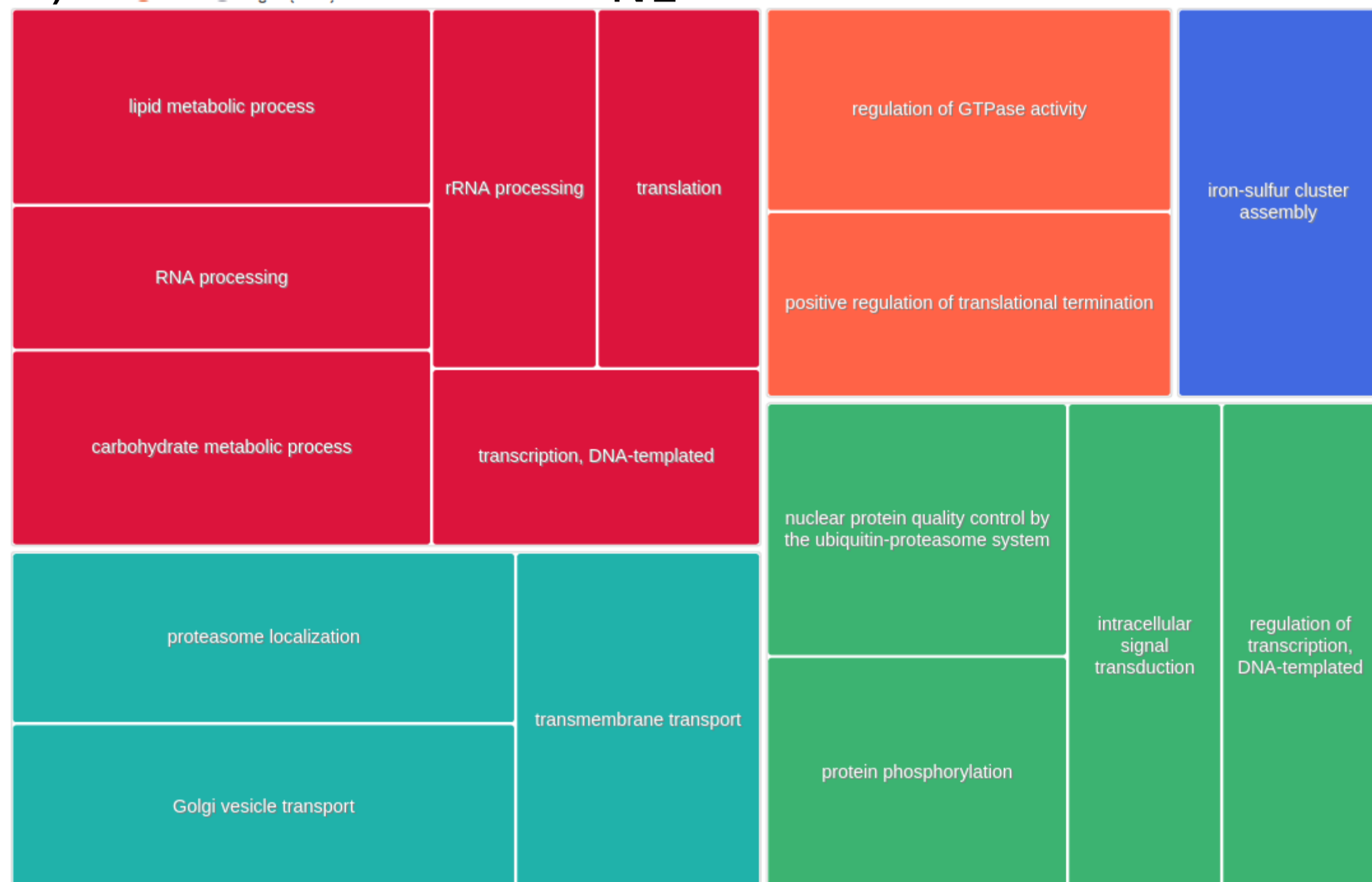

Supplement: Supplementary file 11 — Additional file 11. REVIGO summary of Gene Ontology annotations for genes interested by ancestral insertions. Each rectangle is a single cluster representative. The representatives are joined into 'superclusters' of loosely related terms, visualized with different colors. Rectangles are sized relatively based on how many GO terms were found in a category. [file 43008_2023_128_MOESM11_ESM.pdf]

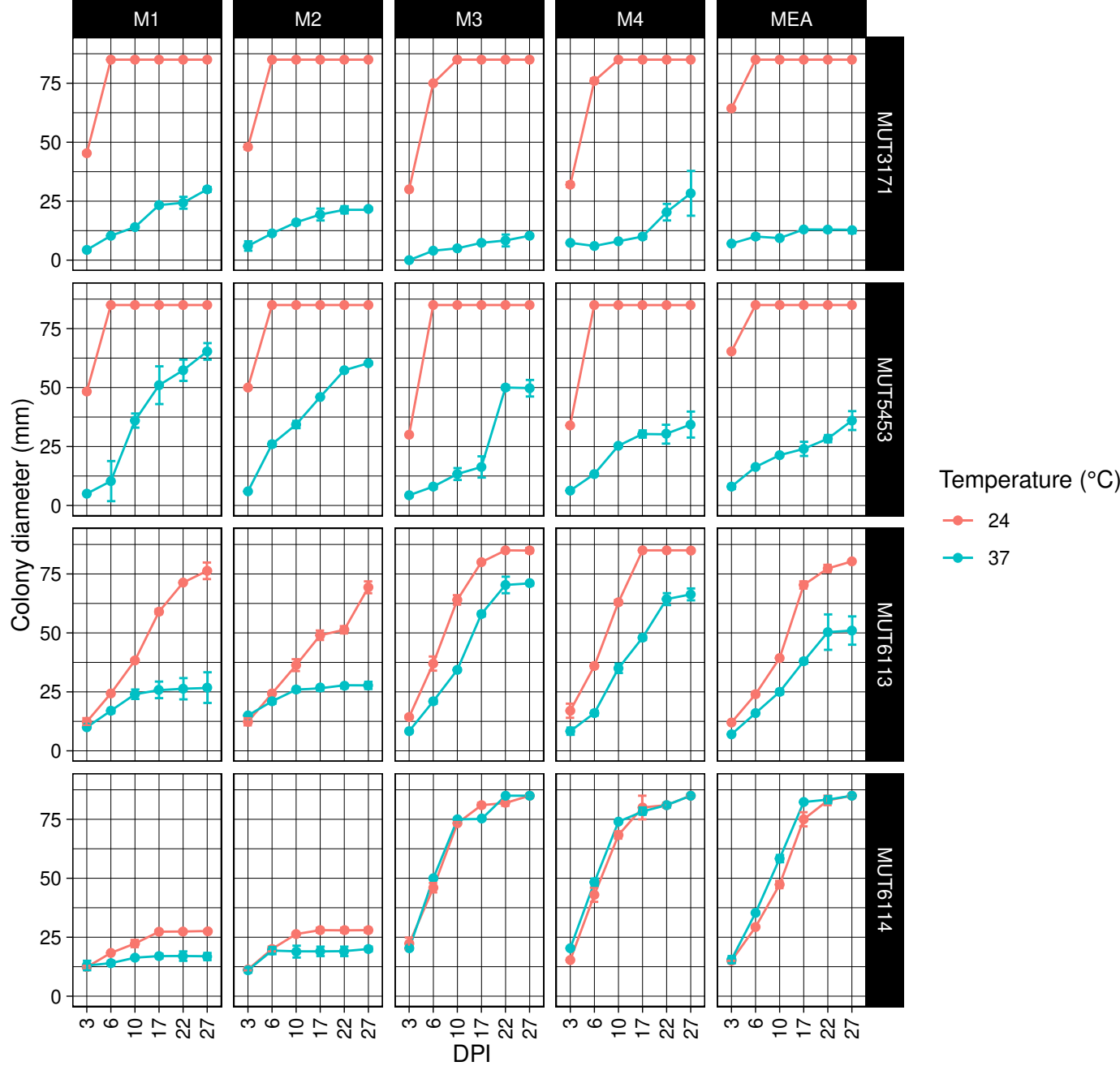

Supplement: Supplementary file 14 — Additional file 14. Graphical representation of the growth rates of S. aurantiacum MUT6114, S. minutisporum MUT6113, T. lixii MUT4171 and T. harzianum MUT5453 on different growth media, at 24 ºC and 37 ºC. In each box, dots are distributed on the vertical axis depending on the diameter of the colonies at a specific time point (X axis). The lines, dots and error bars are colored based on the growth temperature. M1 to M4 indicate the different agarized media. [file 43008_2023_128_MOESM14_ESM.pdf]
