# Supplementary material for: The genomes of Scedosporium between environmental challenges and opportunism
Source: IMA Fungus. 2023 Dec 4;14:25. doi: 10.1186/s43008-023-00128-3 (PMC10694956; doi:10.1186/s43008-023-00128-3)
Supplement: Supplementary file 10 — Additional file 10. Genome architecture in Microascaceagraphical representation of the growth rates of S. aurantiacum MUT6114, S. minutisporum MUT6113, T. lixii MUT4171 and T. harzianum MUT5453 on different growth media, at 24 °C and 37 °C. In each box, dots are distributed on the vertical axis depending on the diameter of the colonies at a specific time point (X axis). The lines, dots and error bars are colored based on the growth temperature. M1 to M4 indicate the different agarized media. e. The flanking distance between neighboring genes provides a measurement of local gene density and is displayed as a colour-coded heatmap, based on a whole-genome analysis. The graphics are organized in bins: each area is colored with more or less intensity based on how many genes in the genome have that 5’ (Y axis) and 3’ (X axis) distances with their neighbors. The majority of genes in Microascaceae are found in gene-dense regions (contrary to two-speed genomes). The distribution of ancestral (N0 and N1) and strain-specific insertions is also displayed with different shapes, revealing a topological overlap between TEs and gene space. [file 43008_2023_128_MOESM10_ESM.pdf]

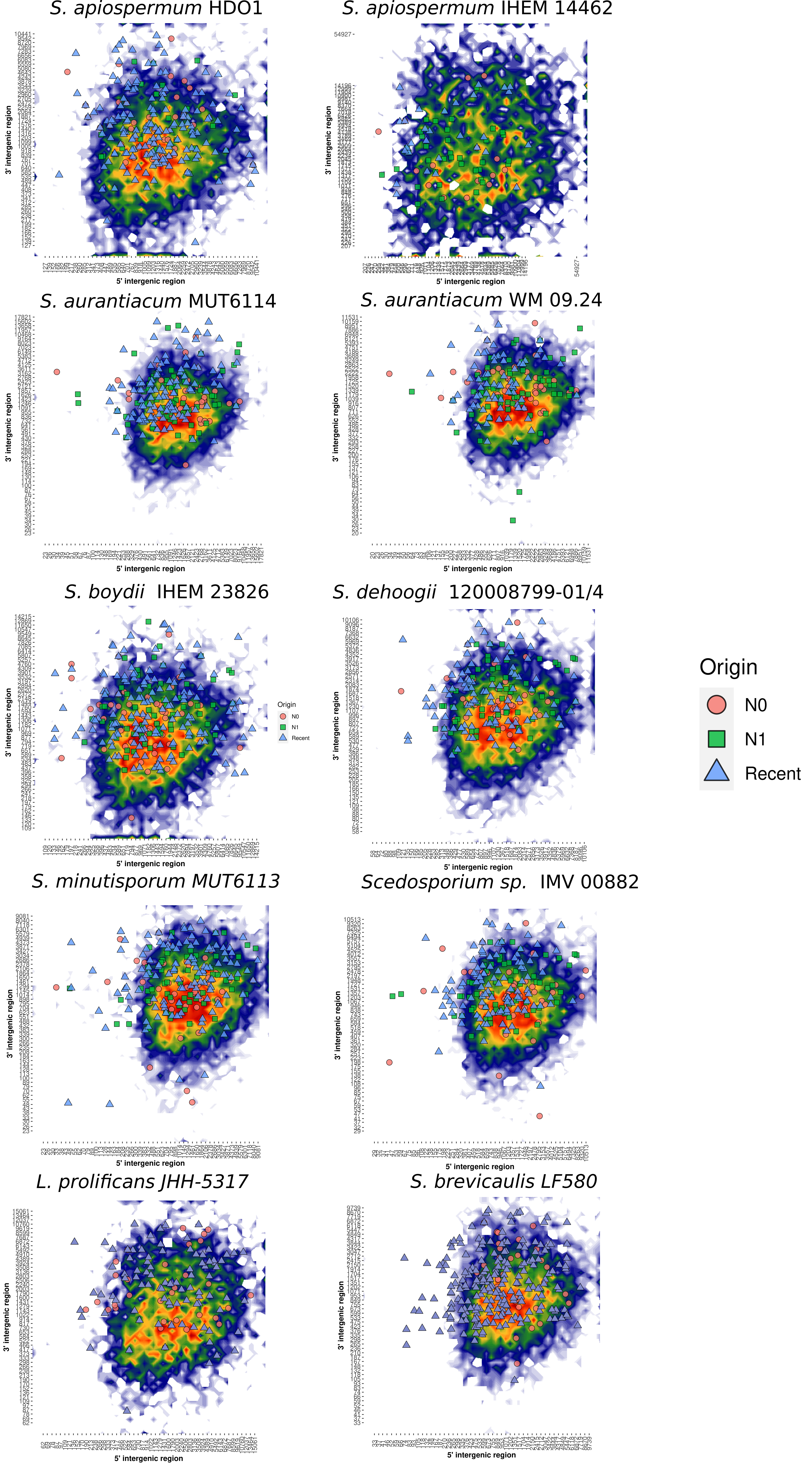
S. apiospermum HDO1
S. apiospermum IHEM 14462
S. aurantiacum MUT6114
S. aurantiacum WM 09.24
S. boydii IHEM 23826
S. dehoogii 120008799-01/4
S. minutisporum MUT6113
Scedosporium sp. IMV 00882
L. prolificans JHH-5317
S. brevicaulis LF580
3' intergenic region
5' intergenic region
Origin
N0
N1
Recent
