# Supplementary material for: The genomes of Scedosporium between environmental challenges and opportunism
Source: IMA Fungus. 2023 Dec 4;14:25. doi: 10.1186/s43008-023-00128-3 (PMC10694956; doi:10.1186/s43008-023-00128-3)
Supplement: Supplementary file 13 — Additional file 13 (a–k). REVIGO summary of Gene Ontology annotations for genes interested by strain-specific insertions. Each rectangle is a single cluster representative. The representatives are joined into 'superclusters' of loosely related terms, visualized with different colors. Rectangles are sized relatively based on how many GO terms were found in a category. [file 43008_2023_128_MOESM13_ESM.pdf]

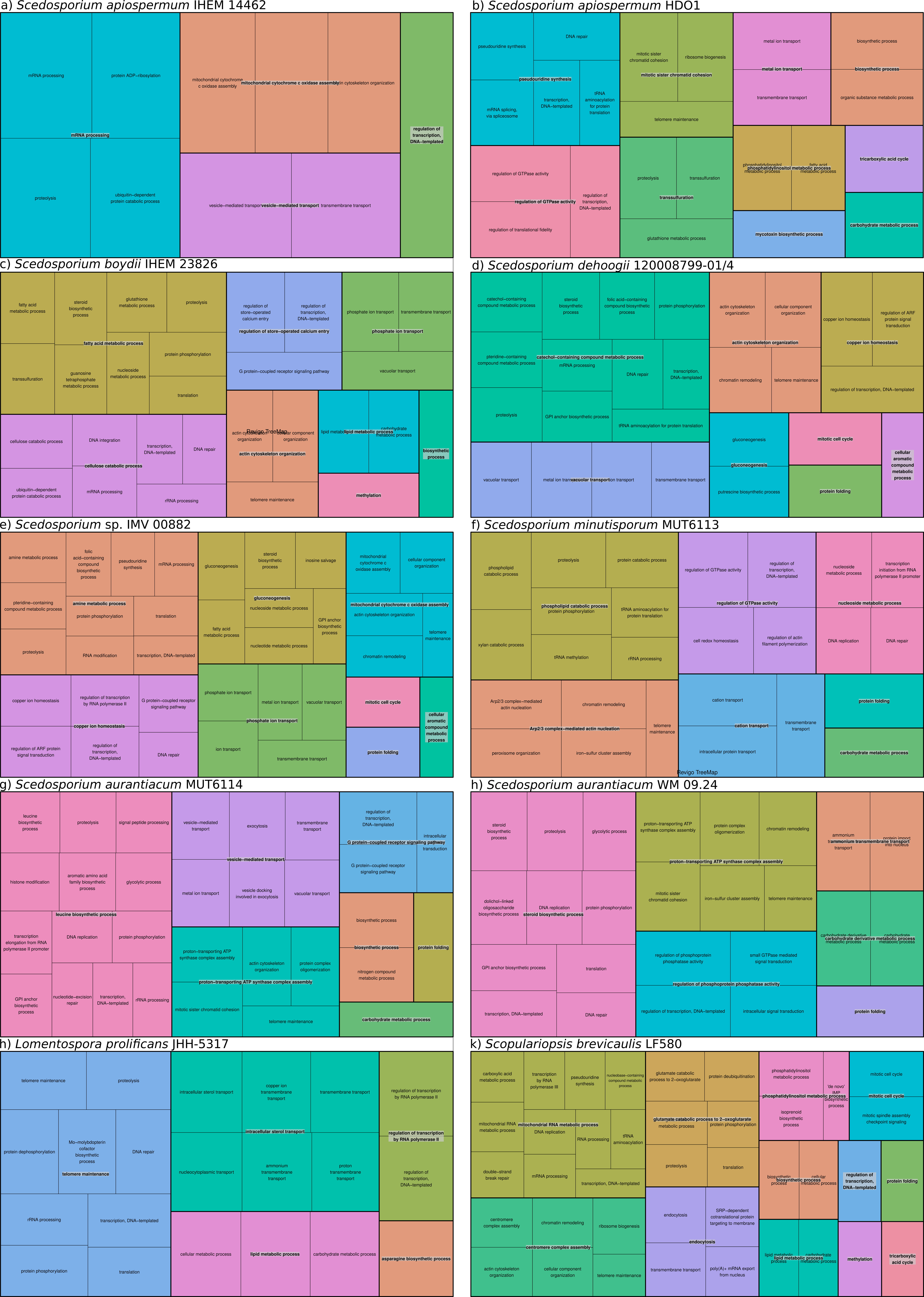
a) Scedosporium apiospermum IHEM 14462
mRNA processing
protein ADP−ribosylation
mitochondrial cytochrome c oxidase assembly
mitochondrial cytochrome c oxidase assembly
actin cytoskeleton organization
regulation of transcription, DNA−templated
proteolysis
ubiquitin−dependent protein catabolic process
mRNA processing
vesicle−mediated transport
vesicle−mediated transport
transmembrane transport
b) Scedosporium apiospermum HDO1
pseudouridine synthesis
DNA repair
mitotic sister chromatid cohesion
ribosome biogenesis
metal ion transport
biosynthetic process
pseudouridine synthesis
mitotic sister chromatid cohesion
metal ion transport
biosynthetic process
mRNA splicing, via spliceosome
transcription, DNA−templated
tRNA aminoacylation for protein translation
telomere maintenance
transmembrane transport
organic substance metabolic process
regulation of GTPase activity
proteolysis
transsulfuration
phosphatidylinositol metabolic process
fatty acid metabolic process
phosphatidylinositol metabolic process
tricarboxylic acid cycle
regulation of GTPase activity
regulation of transcription, DNA−templated
transsulfuration
regulation of translational fidelity
glutathione metabolic process
mycotoxin biosynthetic process
carbohydrate metabolic process
c) Scedosporium boydii IHEM 23826
fatty acid metabolic process
steroid biosynthetic process
glutathione metabolic process
proteolysis
regulation of store−operated calcium entry
regulation of transcription, DNA−templated
phosphate ion transport
transmembrane transport
fatty acid metabolic process
regulation of store−operated calcium entry
phosphate ion transport
transsulfuration
guanosine tetraphosphate metabolic process
nucleoside metabolic process
protein phosphorylation
translation
G protein−coupled receptor signaling pathway
vacuolar transport
cellulose catabolic process
DNA integration
transcription, DNA−templated
DNA repair
actin cytoskeleton organization
cellular component organization
lipid metabolic process
carbohydrate metabolic process
biosynthetic process
Revigo TreeMap
cellulose catabolic process
actin cytoskeleton organization
ubiquitin−dependent protein catabolic process
mRNA processing
rRNA processing
telomere maintenance
methylation
d) Scedosporium dehoogii 120008799-01/4
catechol−containing compound metabolic process
steroid biosynthetic process
folic acid−containing compound biosynthetic process
protein phosphorylation
actin cytoskeleton organization
cellular component organization
copper ion homeostasis
regulation of ARF protein signal transduction
actin cytoskeleton organization
copper ion homeostasis
pteridine−containing compound metabolic process
catechol−containing compound metabolic process
mRNA processing
DNA repair
transcription, DNA−templated
chromatin remodeling
telomere maintenance
regulation of transcription, DNA−templated
proteolysis
GPI anchor biosynthetic process
tRNA aminoacylation for protein translation
gluconeogenesis
mitotic cell cycle
cellular aromatic compound metabolic process
gluconeogenesis
vacuolar transport
metal ion transport
vacuolar transport
transmembrane transport
putrescine biosynthetic process
protein folding
e) Scedosporium sp. IMV 00882
amine metabolic process
folic acid−containing compound biosynthetic process
pseudouridine synthesis
mRNA processing
gluconeogenesis
steroid biosynthetic process
inosine salvage
mitochondrial cytochrome c oxidase assembly
cellular component organization
gluconeogenesis
nucleoside metabolic process
mitochondrial cytochrome c oxidase assembly
actin cytoskeleton organization
pteridine−containing compound metabolic process
amine metabolic process
protein phosphorylation
translation
fatty acid metabolic process
GPI anchor biosynthetic process
telomere maintenance
nucleotide metabolic process
chromatin remodeling
proteolysis
RNA modification
transcription, DNA−templated
copper ion homeostasis
regulation of transcription by RNA polymerase II
G protein−coupled receptor signaling pathway
phosphate ion transport
metal ion transport
vacuolar transport
mitotic cell cycle
cellular aromatic compound metabolic process
copper ion homeostasis
phosphate ion transport
regulation of ARF protein signal transduction
regulation of transcription, DNA−templated
DNA repair
ion transport
transmembrane transport
protein folding
f) Scedosporium minutisporum MUT6113
phospholipid catabolic process
proteolysis
protein catabolic process
regulation of GTPase activity
regulation of transcription, DNA−templated
nucleoside metabolic process
transcription initiation from RNA polymerase II promoter
phospholipid catabolic process
protein phosphorylation
tRNA aminoacylation for protein translation
regulation of GTPase activity
nucleoside metabolic process
xylan catabolic process
tRNA methylation
rRNA processing
cell redox homeostasis
regulation of actin filament polymerization
DNA replication
DNA repair
Arp2/3 complex−mediated actin nucleation
chromatin remodeling
telomere maintenance
cation transport
transmembrane transport
protein folding
Arp2/3 complex−mediated actin nucleation
cation transport
peroxisome organization
iron−sulfur cluster assembly
intracellular protein transport
carbohydrate metabolic process
Revigo TreeMap
g) Scedosporium aurantiacum MUT6114
leucine biosynthetic process
proteolysis
signal peptide processing
vesicle−mediated transport
exocytosis
transmembrane transport
regulation of transcription, DNA−templated
intracellular signal transduction
G protein−coupled receptor signaling pathway
vesicle−mediated transport
histone modification
aromatic amino acid family biosynthetic process
glycolytic process
metal ion transport
vesicle docking involved in exocytosis
vacuolar transport
G protein−coupled receptor signaling pathway
leucine biosynthetic process
biosynthetic process
transcription elongation from RNA polymerase II promoter
DNA replication
protein phosphorylation
proton−transporting ATP synthase complex assembly
actin cytoskeleton organization
protein complex oligomerization
biosynthetic process
protein folding
proton−transporting ATP synthase complex assembly
nitrogen compound metabolic process
GPI anchor biosynthetic process
nucleotide−excision repair
transcription, DNA−templated
rRNA processing
mitotic sister chromatid cohesion
telomere maintenance
carbohydrate metabolic process
h) Scedosporium aurantiacum WM 09.24
steroid biosynthetic process
proteolysis
glycolytic process
proton−transporting ATP synthase complex assembly
protein complex oligomerization
chromatin remodeling
ammonium transmembrane transport
ammonium transmembrane transport
protein import into nucleus
proton−transporting ATP synthase complex assembly
dolichol−linked oligosaccharide biosynthetic process
DNA replication
steroid biosynthetic process
protein phosphorylation
mitotic sister chromatid cohesion
iron−sulfur cluster assembly
telomere maintenance
carbohydrate derivative metabolic process
carbohydrate derivative metabolic process
carbohydrate metabolic process
regulation of phosphoprotein phosphatase activity
small GTPase mediated signal transduction
GPI anchor biosynthetic process
translation
regulation of phosphoprotein phosphatase activity
transcription, DNA−templated
DNA repair
regulation of transcription, DNA−templated
intracellular signal transduction
protein folding
h) Lomentospora prolificans JHH-5317
telomere maintenance
proteolysis
intracellular sterol transport
copper ion transmembrane transport
transmembrane transport
regulation of transcription by RNA polymerase II
intracellular sterol transport
regulation of transcription by RNA polymerase II
protein dephosphorylation
Mo−molybdopterin cofactor biosynthetic process
telomere maintenance
DNA repair
nucleocytoplasmic transport
ammonium transmembrane transport
proton transmembrane transport
regulation of transcription, DNA−templated
rRNA processing
transcription, DNA−templated
protein phosphorylation
translation
cellular metabolic process
lipid metabolic process
carbohydrate metabolic process
asparagine biosynthetic process
k) Scopulariopsis brevicaulis LF580
carboxylic acid metabolic process
transcription by RNA polymerase III
pseudouridine synthesis
nucleobase−containing compound metabolic process
glutamate catabolic process to 2−oxoglutarate
protein deubiquitination
phosphatidylinositol metabolic process
'de novo' IMP biosynthetic process
mitotic cell cycle
phosphatidylinositol metabolic process
mitotic cell cycle
mitochondrial RNA metabolic process
mitochondrial RNA metabolic process
DNA replication
RNA processing
tRNA aminoacylation
glutamate catabolic process to 2−oxoglutarate
metabolic process
protein phosphorylation
isoprenoid biosynthetic process
mitotic spindle assembly checkpoint signaling
double−strand break repair
mRNA processing
transcription, DNA−templated
proteolysis
translation
biosynthetic process
biosynthetic process
cellular metabolic process
regulation of transcription, DNA−templated
protein folding
centromere complex assembly
chromatin remodeling
ribosome biogenesis
endocytosis
SRP−dependent cotranslational protein targeting to membrane
endocytosis
lipid metabolic process
lipid metabolic process
carbohydrate metabolic process
methylation
tricarboxylic acid cycle
centromere complex assembly
actin cytoskeleton organization
cellular component organization
telomere maintenance
transmembrane transport
poly(A)+ mRNA export from nucleus
