# Supplementary material for: The genomes of Scedosporium between environmental challenges and opportunism
Source: IMA Fungus. 2023 Dec 4;14:25. doi: 10.1186/s43008-023-00128-3 (PMC10694956; doi:10.1186/s43008-023-00128-3)

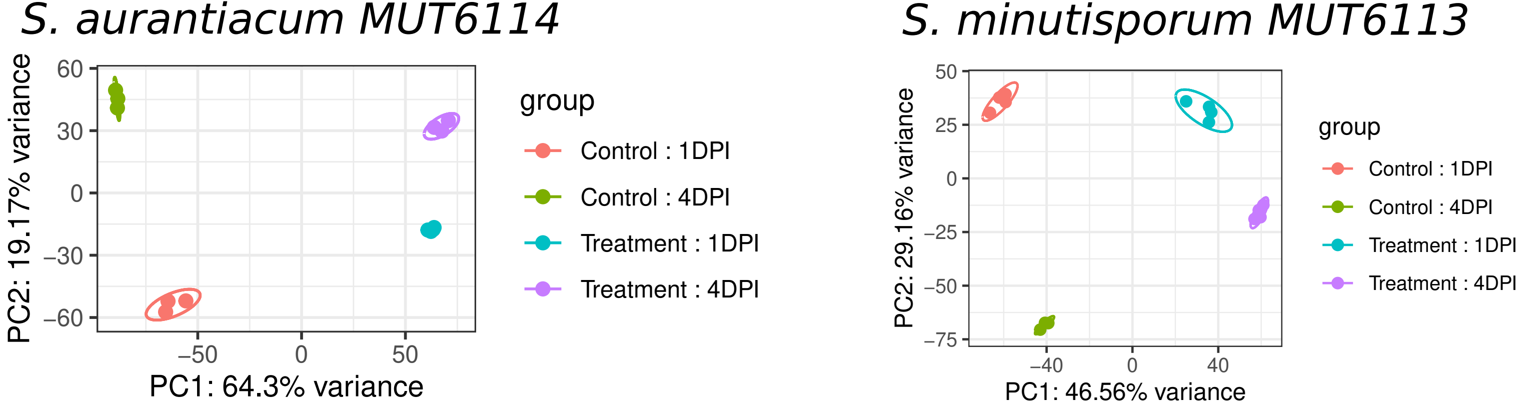

	1 DPI				4 DPI			
	N. of DEGs	% of DEGs (over all genes)	% up-regulated	% down-regulated	N. of DEGs	% of DEGs (over all genes)	% up-regulated	% down-regulated
S. aurantiacum MUT6114	6041	56	51.4	48.6	6838	63.4	50.3	49.7
S. minutisporum MUT6113	5915	47.4	49.6	50.4	5819	47.1	47.8	52.2

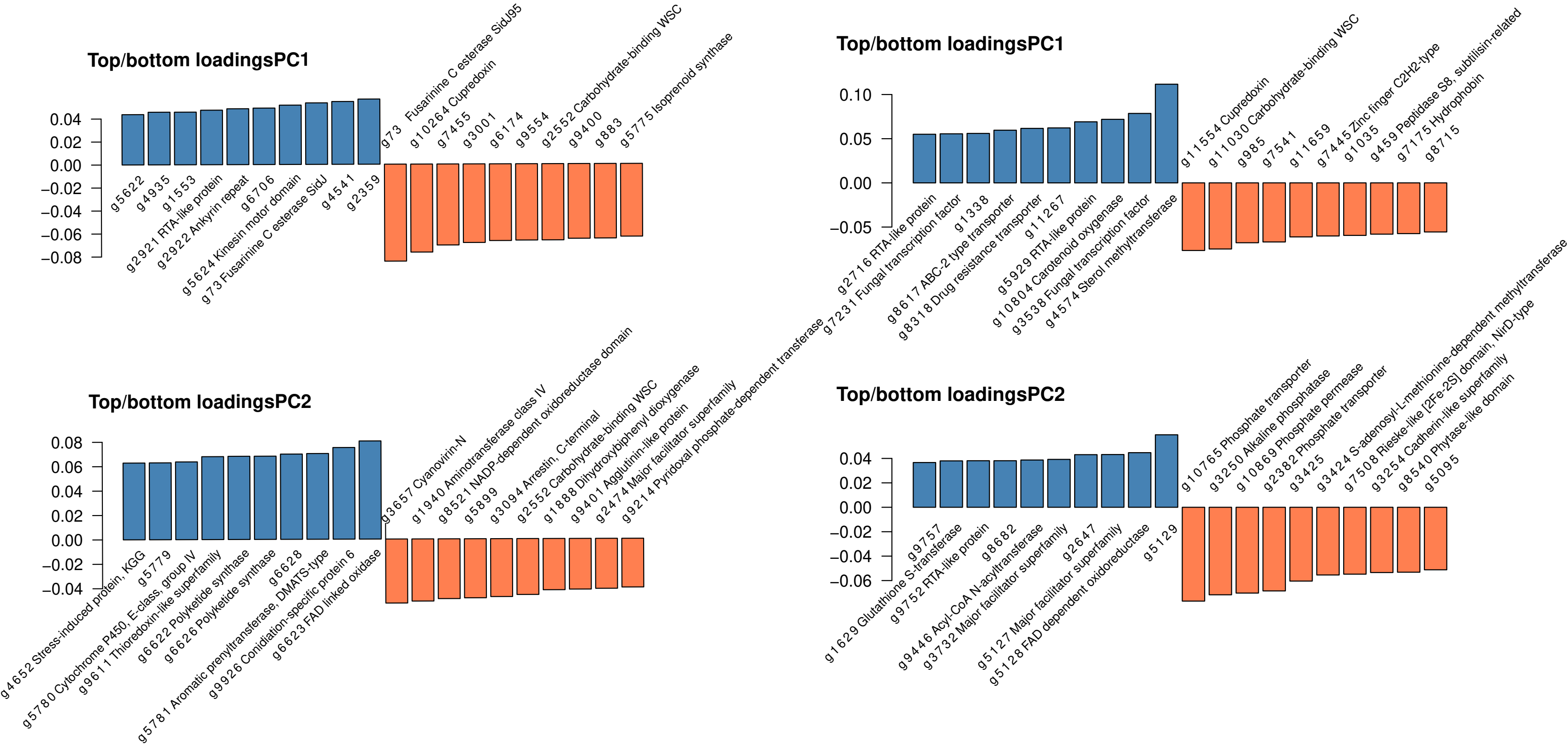

Supplement: Supplementary file 15 — Additional file 15. Summary of the transcriptome for S. aurantiacum MUT6114 and S. minutisporum MUT6113 in response to voriconazole. Principal Components Analyses (PCA) and a summary table are viewed at the top of the figure. The bottom area shows, for each strain and time point, the bottom and top loadings for PCA, i.e. the genes that mostly influence the distribution of dots on PC1 and PC2, and their annotations. [file 43008_2023_128_MOESM15_ESM.pdf]
